# Association Between Tumor Necrosis Factor Inhibitors and the Risk of Hospitalization or Death Among Patients With Immune-Mediated Inflammatory Disease and COVID-19

**DOI:** 10.1001/jamanetworkopen.2021.29639

**Published:** 2021-10-18

**Authors:** Zara Izadi, Erica J. Brenner, Satveer K. Mahil, Nick Dand, Zenas Z. N. Yiu, Mark Yates, Ryan C. Ungaro, Xian Zhang, Manasi Agrawal, Jean-Frederic Colombel, Milena A. Gianfrancesco, Kimme L. Hyrich, Anja Strangfeld, Loreto Carmona, Elsa F. Mateus, Saskia Lawson-Tovey, Eva Klingberg, Giovanna Cuomo, Marta Caprioli, Ana Rita Cruz-Machado, Ana Carolina Mazeda Pereira, Rebecca Hasseli, Alexander Pfeil, Hanns-Martin Lorenz, Bimba Franziska Hoyer, Laura Trupin, Stephanie Rush, Patricia Katz, Gabriela Schmajuk, Lindsay Jacobsohn, Andrea M. Seet, Samar Al Emadi, Leanna Wise, Emily L. Gilbert, Alí Duarte-García, Maria O. Valenzuela-Almada, Carolina A. Isnardi, Rosana Quintana, Enrique R. Soriano, Tiffany Y-T. Hsu, Kristin M. D’Silva, Jeffrey A. Sparks, Naomi J. Patel, Ricardo Machado Xavier, Claudia Diniz Lopes Marques, Adriana Maria Kakehasi, René-Marc Flipo, Pascal Claudepierre, Alain Cantagrel, Philippe Goupille, Zachary S. Wallace, Suleman Bhana, Wendy Costello, Rebecca Grainger, Jonathan S. Hausmann, Jean W. Liew, Emily Sirotich, Paul Sufka, Philip C. Robinson, Pedro M. Machado, Christopher E. M. Griffiths, Jonathan N. Barker, Catherine H. Smith, Jinoos Yazdany, Michael D. Kappelman

**Affiliations:** 1 Department of Epidemiology and Biostatistics, University of California, San Francisco, San Francisco; 2 Division of Rheumatology, Department of Medicine, University of California, San Francisco, San Francisco; 3 Division of Pediatric Gastroenterology, Department of Pediatrics, University of North Carolina at Chapel Hill, Chapel Hill; 4 Guy’s and St Thomas’ NHS Foundation Trust, London, United Kingdom; 5 St John’s Institute of Dermatology, King’s College London, London, United Kingdom; 6 Department of Medical and Molecular Genetics, School of Basic and Medical Biosciences, Faculty of Life Sciences and Medicine, King’s College London, London, United Kingdom; 7 Health Data Research UK, London, United Kingdom; 8 Dermatology Centre, The University of Manchester, Manchester Academic Health Science Centre, NIHR Manchester Biomedical Research Centre, Manchester, United Kingdom; 9 Salford Royal NHS Foundation Trust, Pendleton, Salford, England; 10 Centre for Rheumatic Diseases, King’s College London, London, United Kingdom; 11 Henry D. Janowitz Division of Gastroenterology, Icahn School of Medicine at Mount Sinai, New York, New York; 12 Division of Gastroenterology, Department of Medicine, University of North Carolina at Chapel Hill, Chapel Hill; 13 Centre for Epidemiology Versus Arthritis, The University of Manchester, Manchester Academic Health Science Centre, Manchester, United Kingdom; 14 NIHR Manchester Biomedical Research Centre, The University of Manchester, Manchester Academic Health Science Centre, Manchester, United Kingdom; 15 Manchester University NHS Foundation Trust, Manchester, United Kingdom; 16 Epidemiology and Health Care Research, German Rheumatism Research Center, Berlin, Germany; 17 Instituto de Salud Musculoesquelética, Madrid, Spain; 18 Portuguese League Against Rheumatic Diseases, Lisbon, Portugal; 19 European League Against Rheumatism Standing Committee of People With Arthritis/Rheumatism in Europe, Kilchberg, Switzerland; 20 Centre for Genetics and Genomics Versus Arthritis, Centre for Musculoskeletal Research, The University of Manchester, Manchester, United Kingdom; 21 Department of Rheumatology and Inflammation Research, Sahlgrenska Academy at the University of Gothenburg, Sweden; 22 Department of Precision Medicine, University of Campania Luigi Vanvitelli, Napoli, Italy; 23 Istituto di Ricovero e Cura a Carattere Scientifico, Humanitas Research Hospital, Milan, Italy; 24 Rheumatology Department, Hospital de Santa Maria, CHULN, Lisbon Academic Medical Centre, Lisbon, Portugal; 25 Rheumatology Research Unit, Instituto de Medicina Molecular João Lobo Antunes, Faculdade de Medicina, Universidade de Lisboa, Lisbon, Portugal; 26 Rheumatology Department, Centro Hospitalar do Baixo Vouga, Aveiro, Portugal; 27 Department of Rheumatology and Clinical Immunology, Campus Kerckhoff, Justus-Liebig-University, Giessen, Germany; 28 Department of Internal Medicine, Jena University Hospital, Friedrich Schiller University Jena, Jena, Germany; 29 Division of Rheumatology, Department of Medicine, University Hospital Heidelberg, Heidelberg, Germany; 30 German Society for Rheumatology, Berlin, Germany; 31 University Medical Center Schleswig-Holstein, Kiel, Germany; 32 San Francisco VA Healthcare System, San Francisco, California; 33 Rheumatology Department, Hamad Medical Corporation, Doha, Qatar; 34 Division of Rheumatology, Department of Internal Medicine, University of Southern California, Los Angeles; 35 Division of Rheumatology, Mayo Clinic, Jacksonville, Florida; 36 Division of Rheumatology, Mayo Clinic, Rochester, Minnesota; 37 Robert D. and Patricia E. Kern Center for the Science of Health Care, Mayo Clinic, Rochester, Minnesota; 38 Argentine Society of Rheumatology, Buenos Aires, Argentina; 39 Rheumatology Unit, Internal Medicine Service, Hospital Italiano de Buenos Aires and Instituto Universitario Hospital Italiano de Buenos Aires, Buenos Aires, Argentina; 40 Division of Rheumatology, Inflammation, and Immunity, Brigham and Women’s Hospital, Boston, Massachusetts; 41 Harvard Medical School, Boston, Massachusetts; 42 Division of Rheumatology, Allergy, and Immunology, Clinical Epidemiology Program, Mongan Institute, Department of Medicine, Massachusetts General Hospital, Boston, Massachusetts; 43 Rheumatology Unit, Division of Rheumatology, Allergy, and Immunology, Massachusetts General Hospital, Boston, Massachusetts; 44 Hospital de Clínicas, Federal University of Rio Grande do Sul, Porto Alegre, Brazil; 45 Hospital das Clínicas, Federal University of Pernambuco, Recife, Brazil; 46 Hospital das Clínicas, Federal University of Minas Gerais, Belo Horizonte, Brazil.; 47 Department of Rheumatology, University of Lille, Lille, France; 48 EpiDermE, Université Paris Est Créteil, Créteil, France; 49 Rheumatology Department, Henri-Mondor University Hospital, Créteil, France; 50 Toulouse University Hospital, Toulouse, France; 51 Rheumatology Department, Chru Hospitals of Tours, Tours, France; 52 Groupe Innovation and Ciblage Cellulaire, University of Tours, Tours, France; 53 Crystal Run Health, Middletown, New York; 54 Irish Children’s Arthritis Network, Bansha, Tipperary, Ireland; 55 Department of Medicine, University of Otago, Wellington, New Zealand; 56 Rheumatology Program, Boston Children’s Hospital, Boston, Massachusetts; 57 Division of Rheumatology and Clinical Immunology, Beth Israel Deaconess Medical Center, Harvard Medical School, Boston, Massachusetts; 58 Section of Rheumatology, Department of Medicine, Boston University School of Medicine, Boston, Massachusetts; 59 Department of Health Research Methods, Evidence, and Impact, McMaster University, Hamilton, Ontario, Canada; 60 Canadian Arthritis Patient Alliance, Toronto, Ontario, Canada; 61 HealthPartners Specialty Center–Rheumatology, St. Paul, Minnesota; 62 Faculty of Medicine, University of Queensland, Brisbane, Australia; 63 Royal Brisbane and Women’s Hospital, Metro North Hospital and Health Service, Queensland, Australia; 64 Centre for Rheumatology and Department of Neuromuscular Diseases, University College London, London, United Kingdom; 65 NIHR University College London Hospitals Biomedical Research Centre, University College London Hospitals, NHS Foundation Trust, London, United Kingdom; 66 Department of Rheumatology, Northwick Park Hospital, London North West University Healthcare NHS Trust, London, United Kingdom; 67 St John’s Institute of Dermatology, Faculty of Life Sciences and Medicine, King’s College London, London, United Kingdom

## Abstract

**Importance:**

Although tumor necrosis factor (TNF) inhibitors are widely prescribed globally because of their ability to ameliorate shared immune pathways across immune-mediated inflammatory diseases (IMIDs), the impact of COVID-19 among individuals with IMIDs who are receiving TNF inhibitors remains insufficiently understood.

**Objective:**

To examine the association between the receipt of TNF inhibitor monotherapy and the risk of COVID-19–associated hospitalization or death compared with other commonly prescribed immunomodulatory treatment regimens among adult patients with IMIDs.

**Design, Setting, and Participants:**

This cohort study was a pooled analysis of data from 3 international COVID-19 registries comprising individuals with rheumatic diseases, inflammatory bowel disease, and psoriasis from March 12, 2020, to February 1, 2021. Clinicians directly reported COVID-19 outcomes as well as demographic and clinical characteristics of individuals with IMIDs and confirmed or suspected COVID-19 using online data entry portals. Adults (age ≥18 years) with a diagnosis of inflammatory arthritis, inflammatory bowel disease, or psoriasis were included.

**Exposures:**

Treatment exposure categories included TNF inhibitor monotherapy (reference treatment), TNF inhibitors in combination with methotrexate therapy, TNF inhibitors in combination with azathioprine/6-mercaptopurine therapy, methotrexate monotherapy, azathioprine/6-mercaptopurine monotherapy, and Janus kinase (Jak) inhibitor monotherapy.

**Main Outcomes and Measures:**

The main outcome was COVID-19–associated hospitalization or death. Registry-level analyses and a pooled analysis of data across the 3 registries were conducted using multilevel multivariable logistic regression models, adjusting for demographic and clinical characteristics and accounting for country, calendar month, and registry-level correlations.

**Results:**

A total of 6077 patients from 74 countries were included in the analyses; of those, 3215 individuals (52.9%) were from Europe, 3563 individuals (58.6%) were female, and the mean (SD) age was 48.8 (16.5) years. The most common IMID diagnoses were rheumatoid arthritis (2146 patients [35.3%]) and Crohn disease (1537 patients [25.3%]). A total of 1297 patients (21.3%) were hospitalized, and 189 patients (3.1%) died. In the pooled analysis, compared with patients who received TNF inhibitor monotherapy, higher odds of hospitalization or death were observed among those who received a TNF inhibitor in combination with azathioprine/6-mercaptopurine therapy (odds ratio [OR], 1.74; 95% CI, 1.17-2.58; *P* = .006), azathioprine/6-mercaptopurine monotherapy (OR, 1.84; 95% CI, 1.30-2.61; *P* = .001), methotrexate monotherapy (OR, 2.00; 95% CI, 1.57-2.56; *P* < .001), and Jak inhibitor monotherapy (OR, 1.82; 95% CI, 1.21-2.73; *P* = .004) but not among those who received a TNF inhibitor in combination with methotrexate therapy (OR, 1.18; 95% CI, 0.85-1.63; *P* = .33). Similar findings were obtained in analyses that accounted for potential reporting bias and sensitivity analyses that excluded patients with a COVID-19 diagnosis based on symptoms alone.

**Conclusions and Relevance:**

In this cohort study, TNF inhibitor monotherapy was associated with a lower risk of adverse COVID-19 outcomes compared with other commonly prescribed immunomodulatory treatment regimens among individuals with IMIDs.

## Key Points

**Question:**

Is receipt of tumor necrosis factor (TNF) inhibitor monotherapy at the time of COVID-19 diagnosis associated with adverse COVID-19 outcomes compared with other treatment regimens among patients with immune-mediated inflammatory diseases (IMIDs)?

**Findings:**

In this cohort study of 6077 patients with IMIDs and COVID-19, TNF inhibitors in combination with azathioprine/6-mercaptopurine therapy, methotrexate monotherapy, azathioprine/6-mercaptopurine monotherapy, or Janus kinase inhibitor monotherapy were each associated with significantly higher odds of hospitalization or death compared with TNF inhibitor monotherapy.

**Meaning:**

This study’s findings support the continued use of TNF inhibitor monotherapy among individuals with IMIDs during the pandemic.

## Teaser

This cohort study uses data from 3 international registries to examine the association between the receipt of tumor necrosis factor monotherapy and the risk of COVID-19–associated hospitalization or death among adult patients with immune-mediated inflammatory diseases.

## Introduction

Patients with COVID-19, caused by SARS-CoV-2, can have mild symptoms or experience a severe and/or life-threatening infection.^1^ Comorbidities, such as lung disease, diabetes, and obesity, increase the risk of adverse COVID-19 outcomes.^2^ Any benefits of treatments for immune-mediated inflammatory diseases (IMIDs) for COVID-19 outcomes remain a topic of interest. These treatments impact the immune system and are associated with a higher risk of infections overall.^3^ This association raises concern about impaired immune response to SARS-CoV-2 among patients currently receiving treatment for IMIDs. However, many damaging consequences of SARS-CoV-2 infection are produced by a hyperinflammatory response.^4^ Therefore, treatments that target an overactive immune response may have protective benefits against adverse COVID-19 outcomes.^1,4^

Tumor necrosis factor (TNF) inhibitors, a class of biologic therapies that target the proinflammatory cytokine TNF, are first- or second-line treatments for many IMIDs. International registries of patients with IMIDs have provided initial information regarding COVID-19 outcomes among individuals who received TNF inhibitor therapies during the pandemic. An analysis of data from the Secure Epidemiology of Coronavirus Under Research Exclusion for Inflammatory Bowel Disease (SECURE-IBD) registry, which includes patients with inflammatory bowel disease (IBD) who were diagnosed with COVID-19, found that prevalent use compared with no use of TNF inhibitors at COVID-19 diagnosis was not associated with severe COVID-19 (odds ratio [OR], 0.9; 95% CI, 0.4-2.2).^5^ A study of data from the Global Rheumatology Alliance (GRA) physician-reported registry of COVID-19 outcomes among people with rheumatic diseases found that prevalent use compared with no use of TNF inhibitors at COVID-19 diagnosis was associated with lower odds of COVID-19–associated hospitalization (OR, 0.40; 95% CI, 0.19-0.81).^6^ An analysis of data from the Psoriasis Patient Registry for Outcomes, Therapy and Epidemiology of COVID-19 Infection (PsoProtect) also found higher odds of hospitalization among patients treated with nonbiologic systemic therapies compared with biologic therapies, including TNF inhibitors (OR, 2.84; 95% CI, 1.31-6.18).^7^ Although studies of individual registries have provided initial information, they were often underpowered to perform more granular analyses of commonly used medications, such as monotherapy vs combination therapy with immunomodulatory drugs, or analyses of medications that are used less frequently.

Pooling data across registries offers an opportunity to rapidly assess any association between TNF inhibitor therapies and COVID-19 outcomes among individuals with IMIDs and to evaluate the consistency of findings across studies and diseases. We pooled data from these 3 international registries of patients with IBD, psoriasis, and rheumatic diseases to evaluate the association between TNF inhibitor monotherapy and COVID-19–associated hospitalization or death compared with other commonly prescribed immunomodulatory regimens among individuals with IMIDs.

## Methods

### Registry Designs and Approvals

Details of the design of the GRA, SECURE-IBD, and PsoProtect registries have been described previously.^7,8,9,10^ In brief, clinicians and trained staff directly report COVID-19 outcomes as well as demographic and clinical characteristics of individuals with IMIDs who have confirmed or suspected COVID-19 using online data entry portals. Quality is assessed by registry-specific data validation teams who remove all known or potential duplicates and address erroneous or ineligible reports. The GRA and PsoProtect registries contain only limited data; no personal identifiers, with the exception of COVID-19 diagnosis dates, are included. The SECURE-IBD registry follows the safe harbor deidentification standards of the Health Insurance Portability and Accountability Act. The GRA registry was determined to be nonhuman subjects research by the United Kingdom Health Research Authority, the University of Manchester (United Kingdom), and the University of California, San Francisco, and informed consent was therefore not required. For the SECURE-IBD registry, the Office for Human Research Ethics at the University of North Carolina at Chapel Hill determined that storage and analysis of deidentified data did not constitute human subjects research and did not require institutional review board approval or informed consent. Voluntary ethical approval was sought by the PsoProtect registry and granted by the Leeds Research Ethics Committee (United Kingdom), who determined that informed consent was not required because of the use of deidentified data. This study followed the Strengthening the Reporting of Observational Studies in Epidemiology (STROBE) reporting guideline for cohort studies.

### COVID-19 Diagnosis

Among patients with rheumatic disease, a COVID-19 diagnosis was based on polymerase chain reaction (PCR), antibody serology testing, or metagenomic testing; computed tomographic scans; laboratory assays; or symptoms alone. Among patients with IBD, a COVID-19 diagnosis was based on PCR testing, symptoms with confirmatory antibody serology testing, or rapid antigen testing. Among patients with psoriasis, both confirmed and suspected COVID-19 diagnoses were reported; however, information regarding the type of diagnostic testing used was not collected.

### Exposures and Outcome

To obtain sufficient statistical power, each exposure category was required to have more than 250 patients in the pooled analysis. Exposure was defined as a categorical variable that comprised the following categories: TNF inhibitor (including adalimumab, certolizumab pegol, etanercept, golimumab, and infliximab) monotherapy (reference category), TNF inhibitors in combination with methotrexate therapy, TNF inhibitors in combination with azathioprine/6-mercaptopurine therapy, methotrexate monotherapy, azathioprine/6-mercaptopurine monotherapy, and Janus kinase (Jak) inhibitor (including tofacitinib, baricitinib, and upadacitinib) monotherapy. The outcome of interest was hospitalization or death associated with COVID-19.

### Inclusion and Exclusion Criteria

We included adults (age ≥18 years) with a diagnosis of inflammatory arthritis, IBD, or psoriasis who were reported to the GRA, SECURE-IBD, or PsoProtect registries, respectively, from March 12, 2020, to February 1, 2021. Our analysis included reconciled patients only. For the GRA registry, reconciled patients were defined as those with at least 1 of the following recorded outcomes: death, symptoms resolved at the time of data entry, not hospitalized more than 30 days after the initial diagnosis date, hospitalized and discharged, or not at risk of further interventions or death. For the SECURE-IBD and PsoProtect registries, patients were defined as reconciled after a minimum of 7 days or 14 days, respectively, or if sufficient time had passed to observe the disease course through the resolution of acute illness or death.

To limit confounding from other immunomodulatory medications, we excluded patients who received an exposure treatment regimen as well as concomitant immunomodulatory drugs, except when sulfasalazine, mesalamine, hydroxychloroquine or chloroquine, leflunomide, oral budesonide, or glucocorticoids were used as concomitant medications.

### Statistical Analysis

We used descriptive statistics to summarize the demographic and clinical characteristics of the study population. Continuous variables were reported as means with SDs or medians with 25th and 75th percentiles, as appropriate. Categorical variables were reported as numbers with percentages. We performed registry-level analyses and a pooled analysis of data across the 3 registries to estimate independent associations between exposure categories and COVID-19 outcomes. Registry-level effect estimates were reported for exposure categories that included 10 or more patients. Associations were estimated using multilevel multivariable mixed-effects logistic regression analysis and reported as odds ratios (ORs) with 95% CIs. We chose mixed-effects regression analysis for its ability to handle missing data using maximum-likelihood estimation and to fit nested random effects to account for multilevel clustering.^11^

Covariates included in all models were age, sex, current smoking, IMID activity (remission vs active disease, as reported by the clinician), important comorbidities (cardiovascular disease [including coronary artery disease, heart failure, and arrhythmia], diabetes, hypertension, obstructive lung disease [including chronic obstructive pulmonary disease and asthma], interstitial or other chronic lung disease, kidney disease [including chronic kidney insufficiency and end-stage kidney disease], obesity [defined as body mass index ≥30; calculated as weight in kilograms divided by height in meters squared], and cancer, each included as a dichotomous variable), and prednisone-equivalent glucocorticoid dose (included as a continuous variable). For the registry-level analyses, we included the following concomitant medications: sulfasalazine, hydroxychloroquine or chloroquine, and leflunomide for the GRA analysis and mesalamine, sulfasalazine, and oral budesonide for the SECURE-IBD analysis. If any of these concomitant medications were significant confounders (*P* < .05) in the registry-level analyses, they were also included as covariates in the pooled analysis; patients from registries that did not include the respective concomitant medications were assigned to the category of individuals with nonuse of these medications. Registry-level analyses also controlled for disease diagnosis; diagnoses in the GRA registry included rheumatoid arthritis (reference category), psoriatic arthritis, spondyloarthritis, and other inflammatory arthritis or more than 1 type of inflammatory arthritis, and diagnoses in the SECURE-IBD registry included Crohn disease (reference category), ulcerative colitis, and unspecified IBD.

We fitted country-level random effects to account for within-country correlations. To account for changes in COVID-19 treatment and health service use over time, we also fitted random effects for the calendar month of symptom onset (PsoProtect registry) or the calendar month during which the patient was diagnosed (GRA registry) or reported (SECURE-IBD registry). The pooled model also included registry-level random effects accounting for within-registry correlations. The hierarchical order of nested random effects in the pooled model was country followed by time and registry. To improve model fit, we removed influential statistical outliers identified in continuous variables (ie, age and glucocorticoid dose) from the analyses. As a result, 2 patients were removed who received a daily prednisone dose greater than 70 mg.

All analyses were conducted using Stata software, version 16.0 (StataCorp). The threshold for statistical significance was 2-sided *P* < .05.

Rheumatology clinics from 2 large health care systems (Mass General Brigham in Massachusetts and Mayo Clinic in Minnesota and Florida) had processes in place to systematically report all patients with COVID-19 to the GRA registry. To assess the extent of potential reporting bias arising from convenience sampling, ORs were derived after reweighting the covariate distribution of patients in the GRA registry to those of the 2 health care systems using the inverse odds of sampling weights technique,^12^ and those values were compared with the original ORs using standardized difference.^13^ In addition, to assess the robustness of results, a pooled sensitivity analysis was performed after excluding patients with a COVID-19 diagnosis that was based on symptoms alone.

## Results

As of February 1, 2021, 8268 patients were reported to have received an exposure treatment regimen at COVID-19 diagnosis. Of those, 5220 patients were from the GRA registry, 2720 patients were from the SECURE-IBD registry, and 328 patients were from the PsoProtect registry. A total of 6077 patients from 74 countries met study eligibility criteria and were included in the analyses; of those, 3441 patients (56.6%) were from the GRA registry, 2336 patients (38.4%) were from the SECURE-IBD registry, and 300 patients (4.9%) were from the PsoProtect registry. Of the 2191 patients excluded from the analyses, most were excluded because they had a rheumatic disease diagnosis other than inflammatory arthritis (827 patients), were patients who were nonreconciled (581 patients), or received concomitant medications that were listed in the exclusion criteria (551 patients) (Table 1).

**Table 1.** Reasons for Study Exclusion

	No.			
	All patients	GRA registry	SECURE-IBD registry	PsoProtect registry
Total patients using an exposure treatment regimen as of February 1, 2021^a^	8268	5220	2720	328
Patients excluded	2191	1779	384	28
Reason for exclusion				
Age missing or <18 y	230	0	226	4
Nonreconciled^b^	581	581	0	0
Noninflammatory arthritis diagnosis	827	827	NA	NA
Receipt of concomitant medication listed in exclusion criteria^c^	551	370	157	24
Influential statistical outliers^d^	2	1	1	0

Abbreviations: GRA, COVID-19 Global Rheumatology Alliance; PsoProtect, Psoriasis Patient Registry for Outcomes, Therapy and Epidemiology of COVID-19 Infection; SECURE-IBD, Secure Epidemiology of Coronavirus Under Research Exclusion for Inflammatory Bowel Disease.

^a^ Exposure treatment regimens included tumor necrosis factor (TNF) inhibitor monotherapy, TNF inhibitors in combination with methotrexate therapy, TNF inhibitors in combination with azathioprine/6-mercaptopurine therapy, methotrexate monotherapy, azathioprine/6-mercaptopurine monotherapy, and Janus kinase inhibitor monotherapy.

^b^ In the GRA registry, a patient was defined as reconciled if at least 1 of the following criteria were present: deceased, symptoms resolved at the time of data entry, not hospitalized >30 days after the initial diagnosis date, hospitalized and discharged, or not at risk of further interventions or death. In the SECURE-IBD and PsoProtect registries, a patient was defined as reconciled after a minimum of 7 days or 14 days, respectively, or if sufficient time had passed to observe the disease course through resolution of acute illness or death.

^c^ Excluded concomitant medications included any medication with the exception of sulfasalazine, mesalamine, hydroxychloroquine or chloroquine, leflunomide, oral budesonide, or glucocorticoids.

^d^ To improve model fit, influential statistical outliers identified in continuous variables were removed. Two patients were removed who received a daily prednisone dose >70 mg.

The demographic and clinical characteristics of the 6077 patients included in the analysis are shown in Table 2. Most patients were from Europe (3215 individuals [52.9%]) and North America (2015 individuals [33.2%]), and the mean (SD) age was 48.8 (16.5) years; 3563 patients (58.6%) were female, and 2468 patients (40.6%) were male. Race and ethnicity were not addressed in these analyses because information on race was not recorded in the PsoProtect registry, and information on race and ethnicity was not available for all countries in the GRA registry. The most common disease diagnoses were rheumatoid arthritis (2146 patients [35.3%]), Crohn disease (1537 patients [25.3%]), ulcerative colitis (762 patients [12.5%]), and spondyloarthritis (624 patients [10.3%]). The most common comorbidities were hypertension (1360 patients [22.4%]), diabetes (541 patients [8.9%]), obstructive lung disease (430 patients [7.1%]), and cardiovascular disease (388 patients [6.4%]). Current smoking and obesity were substantially more prevalent among patients in the PsoProtect registry (42 patients [14.0%] and 92 patients [30.7%], respectively) compared with those in the GRA registry (153 patients [4.4%] and 673 patients [19.6%]) or the SECURE-IBD registry (100 patients [4.3%] and 385 patients [16.5%]).

**Table 2.** Patient Characteristics and COVID-19 Outcomes

Characteristic or outcome	No. (%)			
	Pooled (N = 6077)	GRA (n = 3441)	SECURE-IBD (n = 2336)	PsoProtect (n = 300)
Age, mean (SD), y	48.8 (16.5)	55.0 (14.4)	39.4 (15.4)	49.9 (12.6)
Sex^a^				
Female	3563 (58.6)	2295 (66.7)	1153 (49.4)	115 (38.3)
Male	2468 (40.6)	1144 (33.2)	1139 (48.8)	185 (61.7)
Unknown	46 (0.8)	2 (0.1)	44 (1.9)	0
Region^a^				
Africa	24 (0.4)	16 (0.5)	7 (0.3)	1 (0.3)
Eastern Mediterranean	191 (3.1)	120 (3.5)	68 (2.9)	3 (1.0)
Europe	3215 (52.9)	1800 (52.3)	1143 (48.9)	272 (90.7)
North America	2015 (33.2)	1066 (31.0)	942 (40.3)	7 (2.3)
South America	502 (8.3)	375 (10.9)	111 (4.8)	16 (5.3)
Southeast Asia	22 (0.4)	8 (0.2)	13 (0.6)	1 (0.3)
Western Pacific	85 (1.4)	56 (1.6)	29 (1.2)	0
Unknown	23 (0.4)	0	23 (1.0)	0
Diagnosis^a^				
Rheumatoid arthritis only	2146 (35.3)	2146 (62.4)	NA	NA
Spondyloarthritis only	624 (10.3)	624 (18.1)	NA	NA
Psoriatic arthritis only	566 (9.3)	566 (16.4)	NA	NA
Other inflammatory arthritis or >1 type of inflammatory arthritis	105 (1.7)	105 (3.1)	NA	NA
Crohn disease	1537 (25.3)	NA	1537 (65.8)	NA
Unspecified inflammatory bowel disease	37 (0.6)	NA	37 (1.6)	NA
Ulcerative colitis	762 (12.5)	NA	762 (32.6)	NA
Psoriasis	300 (4.9)	NA	NA	300 (100)
Disease activity^a^				
Remission	2511 (41.3)	1067 (31.0)	1369 (58.6)	75 (25.0)
Active	2918 (48.0)	1829 (53.2)	864 (37.0)	225 (75.0)
Unknown	648 (10.7)	545 (15.8)	103 (4.4)	0
Exposure treatment regimen^a^				
TNF inhibitor monotherapy	2844 (46.8)	1183 (34.4)	1445 (61.9)	216 (72.0)
TNF inhibitor plus methotrexate	669 (11.0)	575 (16.7)	87 (3.7)	7 (2.3)
TNF inhibitor plus azathioprine/6-mercaptopurine	334 (5.5)	7 (0.2)	327 (14.0)	0
Methotrexate monotherapy	1546 (25.4)	1438 (41.8)	31 (1.3)	77 (25.7)
Azathioprine/6-mercaptopurine monotherapy	398 (6.5)	19 (0.6)	379 (16.2)	0
Jak inhibitor monotherapy	286 (4.7)	219 (6.4)	67 (2.9)	0
Concomitant medication				
Sulfasalazine	294 (4.8)	246 (7.1)	48 (2.1)	NA
Mesalamine	384 (6.3)	NA	384 (16.4)	NA
Oral budesonide	39 (0.6)	NA	39 (1.7)	NA
Leflunomide	212 (3.5)	212 (6.2)	NA	NA
Chloroquine or hydroxychloroquine	316 (5.2)	316 (9.2)	NA	NA
Daily glucocorticoid^a^				
No	114 (1.9)	2650 (77.0)	2212 (94.7)	300 (100)
Yes	5162 (84.9)	683 (19.8)	118 (5.1)	0
Unknown	801 (13.2)	108 (3.1)	6 (0.3)	0
Daily prednisone-equivalent glucocorticoid, median (25th percentile-75th percentile), mg	5 (5.0-10.0)	5 (5.0-7.5)	20.0 (5.0-36.0)	NA
Smoking status^a^				
Never or past	4791 (78.8)	2358 (68.5)	2236 (95.7)	197 (65.7)
Current	295 (4.9)	153 (4.4)	100 (4.3)	42 (14.0)
Unknown	991 (16.3)	930 (27.0)	0	61 (20.3)
BMI^a^				
<30	4877 (80.3)	2768 (80.4)	1951 (83.5)	158 (52.7)
≥30	1150 (18.9)	673 (19.6)	385 (16.5)	92 (30.7)
Unknown	50 (0.8)	0	0	50 (16.7)
Lung disease				
Interstitial	164 (2.7)	134 (3.9)	26 (1.1)	4 (1.3)
Obstructive	430 (7.1)	317 (9.2)	99 (4.2)	14 (4.7)
Cardiovascular disease	388 (6.4)	274 (8.0)	90 (3.9)	24 (8.0)
Diabetes	541 (8.9)	401 (11.7)	80 (3.4)	57 (19.0)
Hypertension	1360 (22.4)	1088 (31.6)	193 (8.3)	79 (26.3)
Kidney disease	120 (2.0)	93 (2.7)	24 (1.0)	3 (1.0)
Cancer	117 (1.9)	91 (2.6)	18 (0.8)	8 (2.7)
Hospitalization status^a^				
Not hospitalized	4649 (76.5)	2396 (69.6)	1996 (85.4)	257 (85.7)
Hospitalized	1297 (21.3)	939 (27.3)	316 (13.5)	42 (14.0)
Unknown	131 (2.2)	106 (3.1)	24 (1.0)	1 (0.3)
Death^a^				
Alive	5845 (96.2)	3266 (94.9)	2282 (97.7)	297 (99.0)
Dead	189 (3.1)	166 (4.8)	20 (0.9)	3 (1.0)
Unknown	43 (0.7)	9 (0.3)	34 (1.5)	0
Presumptive COVID-19 diagnosis^b^	864 (14.2)	752 (21.9)	0	112 (37.3)

Abbreviations: BMI, body mass index (calculated as weight in kilograms divided by height in meters squared); GRA, COVID-19 Global Rheumatology Alliance; Jak, Janus kinase; NA, not applicable; PsoProtect, Psoriasis Patient Registry for Outcomes, Therapy and Epidemiology of COVID-19 Infection; SECURE-IBD, Secure Epidemiology of Coronavirus Under Research Exclusion for Inflammatory Bowel Disease; TNF, tumor necrosis factor.

^a^ Subcategories are mutually exclusive.

^b^ Presumptive diagnosis based on symptoms alone.

The receipt of TNF inhibitor monotherapy was reported in 1183 patients (34.4%) from the GRA registry, 1445 patients (61.9%) from the SECURE-IBD registry, and 216 patients (72.0%) from the PsoProtect registry (Table 2). Methotrexate monotherapy was the most prevalent treatment regimen at COVID-19 diagnosis among patients from the GRA registry (1438 individuals [41.8%]). The receipt of azathioprine/6-mercaptopurine, alone or in combination with a TNF inhibitor, was reported in a small proportion of patients from the GRA registry (26 individuals [0.8%]) and in 0 patients from the PsoProtect registry. The receipt of Jak inhibitor monotherapy was reported in 219 patients (6.4%) from the GRA registry, 67 patients (2.9%) from the SECURE-IBD registry, and 0 patients from the PsoProtect registry. A total of 1297 patients (21.3%) were hospitalized, and 189 patients (3.1%) died (Table 2). Both hospitalizations and deaths were more common among patients from the GRA registry (939 patients [27.3%] and 166 patients [4.8%], respectively) than the SECURE-IBD registry (316 patients [13.5%] and 20 patients [0.9%]) or the PsoProtect registry (42 patients [14.0%] and 3 patients [1.0%]).

Along with the prespecified covariates, the concomitant medications sulfasalazine, leflunomide, and oral budesonide were included in the pooled multivariable model because these medications were significantly associated with hospitalization or death in the GRA (sulfasalazine: OR, 1.55 [95% CI, 1.03-2.35; *P* = .04]; leflunomide: OR, 1.97 [95% CI, 1.22-3.18; *P* = .005]) or the SECURE-IBD (oral budesonide: OR, 2.71; 95% CI, 1.11-6.60; *P* = .03) registry-level analyses. In the pooled analysis, compared with TNF inhibitor monotherapy, higher odds of hospitalization or death were observed among those who received a TNF inhibitor in combination with azathioprine/6-mercaptopurine therapy (OR, 1.74; 95% CI, 1.17-2.58; *P* = .006). Differences in the odds of hospitalization or death among those who received TNF inhibitor monotherapy vs a TNF inhibitor in combination with methotrexate therapy were not statistically significant in the registry-specific analyses (GRA: OR, 1.20 [95% CI, 0.80-1.79; *P* = .38]; SECURE-IBD: OR, 1.59 [95% CI, 0.76-3.34; *P* = .22]) or the pooled analysis (OR, 1.18; 95% CI, 0.85-1.63; *P* = .33). Compared with those who received TNF inhibitor monotherapy, higher odds of hospitalization or death were observed among those who received methotrexate monotherapy (OR, 2.00; 95% CI, 1.57-2.56; *P* < .001), azathioprine/6-mercaptopurine monotherapy (OR, 1.84; 95% CI, 1.30-2.61; *P* = .001), and Jak inhibitor monotherapy (OR, 1.82; 95% CI, 1.21-2.73; *P* = .004) in the pooled analysis.

Although ORs obtained from registry-specific analyses were generally in the same direction and of similar extent as those obtained from the pooled analysis, we observed some notable differences (Figure and eTable in Supplement 1). Odds ratios for methotrexate monotherapy compared with TNF inhibitor monotherapy were larger among patients in the PsoProtect registry than patients in the SECURE-IBD or the GRA registries. Odds ratios for azathioprine/6-marcaptopurine monotherapy compared with TNF inhibitor monotherapy were larger among patients in the GRA registry than patients in the SECURE-IBD registry. In addition, the receipt of Jak inhibitor monotherapy was not associated with higher odds of hospitalization or death compared with TNF inhibitor monotherapy (OR, 0.60; 95% CI, 0.22-1.64; *P* = .32) among patients in the SECURE-IBD registry.

**Figure.**
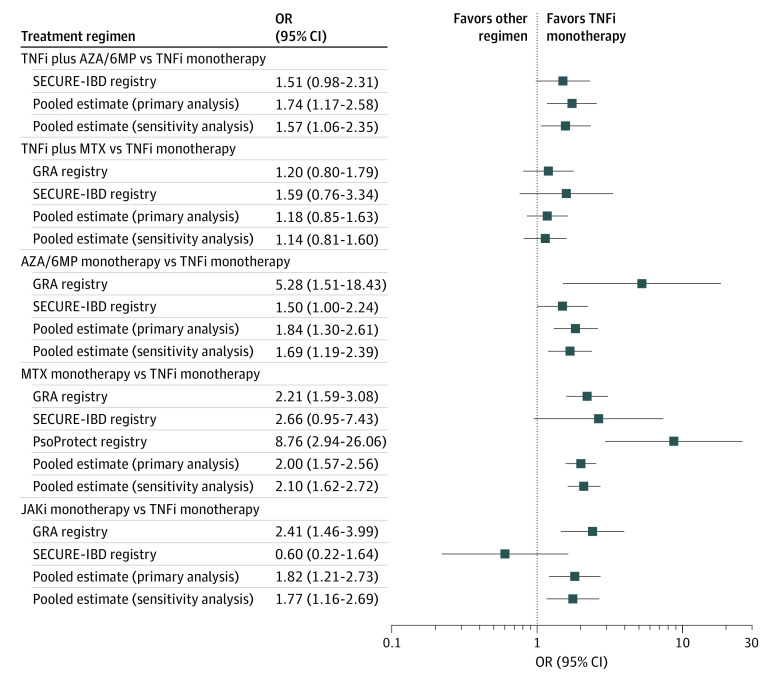
Adjusted Odd Ratios (ORs) of COVID-19–Associated Hospitalization or Death Among Patients Receiving Immunomodulatory Treatment Regimens vs Tumor Necrosis Factor Inhibitor (TNFi) Monotherapy Registry-specific and pooled analyses, with TNFi monotherapy used as the reference category. Pooled estimates were obtained using hierarchical multivariable mixed-effects logistic regression analysis with registry and calendar month random effects nested within country. Pooled sensitivity analysis (n = 5213) excludes patients with a presumptive COVID-19 diagnosis (defined as a diagnosis based on symptoms alone). All ORs were adjusted for age, sex, current smoking, immune-mediated disease activity (remission vs active), important comorbidities (cardiovascular disease, diabetes, hypertension, obstructive lung disease, interstitial or other chronic lung disease, kidney disease, obesity [body mass index ≥30; calculated as weight in kilograms divided by height in meters squared], and cancer), and prednisone-equivalent glucocorticoid dose. The pooled sensitivity analysis was also adjusted for concomitant receipt of leflunomide and oral budesonide. The pooled analysis (N = 6077) was additionally adjusted for concomitant receipt of sulfasalazine. The COVID-19 Global Rheumatology Alliance (GRA) registry-level analysis included 3441 patients and was adjusted for immune-mediated disease diagnosis and concomitant receipt of sulfasalazine, hydroxychloroquine or chloroquine, and leflunomide medications. The Psoriasis Patient Registry for Outcomes, Therapy and Epidemiology of COVID-19 Infection (PsoProtect) registry-level analysis included 300 patients. The Secure Epidemiology of Coronavirus Under Research Exclusion for Inflammatory Bowel Disease (SECURE-IBD) registry-level analysis included 2336 patients and was adjusted for immune-mediated disease diagnosis and concomitant receipt of mesalamine, sulfasalazine, and oral budesonide medications. AZA/6MP indicates azathioprine/6-mercaptopurine; JAKi, Janus kinase inhibitor; and MTX, methotrexate.

Other factors associated with higher odds of hospitalization or death in the pooled analysis included older age (OR per 1 year increase in age, 1.04; 95% CI, 1.04-1.05; *P* < .001); active IMID at COVID-19 diagnosis (OR, 1.27; 95% CI, 1.04-1.55; *P* = .02); obesity (OR, 1.39; 95% CI, 1.10-1.75; *P* = .005); lung disease (interstitial: OR, 1.81 [95% CI, 1.12-2.95; *P* = .02]; obstructive: OR, 2.34 [95% CI, 1.69-3.24; *P* < .001]); cardiovascular disease (OR, 1.58; 95% CI, 1.13-2.21; *P* = .007); diabetes (OR, 1.54; 95% CI, 1.16-2.05; *P* = .003); chronic kidney disease (OR, 3.10; 95% CI, 1.70-5.66; *P* < .001); concomitant use of sulfasalazine (OR, 1.62; 95% CI, 1.13-2.34; *P* = .009), leflunomide (OR, 1.89; 95% CI, 1.20-2.99; *P* = .006), or oral budesonide (OR, 2.86; 95% CI, 1.20-6.84; *P* = .02); and higher daily prednisone-equivalent glucocorticoid dose (OR per 1 mg increase in dose, 1.07; 95% CI, 1.05-1.08; *P* < .001) (Table 3). Female sex was associated with a protective benefit (OR, 0.79; 95% CI, 0.66-0.96; *P* = .02). The intraclass correlation coefficient was 0.27 (95% CI, 0.20-0.36), suggesting that clustering of patients within country, calendar month, and registry explained 27% of the variation in the odds of hospitalization or death. Complete results from registry-specific analyses are shown in the eTable in Supplement 1.

**Table 3.** Adjusted Pooled Odds of COVID-19–Associated Hospitalization or Death Among Patients in the 3 Registries^a^

Variable	OR (95% CI)	*P* value
Exposure treatment regimen^b^		
TNF inhibitor monotherapy	1 [Reference]	NA
TNF inhibitor plus methotrexate	1.18 (0.85-1.63)	.33
TNF inhibitor plus azathioprine/6-mercaptopurine	1.74 (1.17-2.58)	.006
Methotrexate monotherapy	2.00 (1.57-2.56)	<.001
Azathioprine/6-mercaptopurine monotherapy	1.84 (1.30-2.61)	.001
Jak inhibitor monotherapy	1.82 (1.21-2.73)	.004
Concomitant medication		
Sulfasalazine	1.62 (1.13-2.34)	.009
Leflunomide	1.89 (1.20-2.99)	.006
Oral budesonide	2.86 (1.20-6.84)	.02
Daily prednisone-equivalent dose per 1 mg increase	1.07 (1.05-1.08)	<.001
Demographic characteristic		
Female sex	0.79 (0.66-0.96)	.02
Age per year	1.04 (1.04-1.05)	<.001
Obesity (BMI ≥30)	1.39 (1.10-1.75)	.005
Current smoking	0.77 (0.51-1.17)	.21
Disease activity		
Active	1.27 (1.04-1.55)	.02
Comorbidities		
Interstitial lung disease	1.81 (1.12-2.95)	.02
Obstructive lung disease	2.34 (1.69-3.24)	<.001
Cardiovascular disease	1.58 (1.13-2.21)	.007
Diabetes	1.54 (1.16-2.05)	.003
Hypertension	1.19 (0.95-1.50)	.12
Kidney disease	3.10 (1.70-5.66)	<.001
Cancer	1.16 (0.65-2.07)	.61

Abbreviations: BMI, body mass index (calculated as weight in kilograms divided by height in meters squared); Jak, Janus kinase; NA, not applicable; OR, odds ratio; TNF, tumor necrosis factor.

^a^ All 6077 patients from the COVID-19 Global Rheumatology Alliance (GRA); the Psoriasis Patient Registry for Outcomes, Therapy and Epidemiology of COVID-19 Infection (PsoProtect); and the Secure Epidemiology of Coronavirus Under Research Exclusion for Inflammatory Bowel Disease (SECURE-IBD) registries were included.

^b^ Subcategories are mutually exclusive. Odds ratios were obtained using hierarchical multivariable mixed-effects logistic regression analysis with registry and calendar month random effects nested within country. Model was adjusted for all variables shown.

We compared GRA registry-specific results with results obtained after reweighting the covariate distribution of the GRA population to match those of rheumatology clinics that systematically reported all patients diagnosed with COVID-19. Standardized differences between the reweighted and original estimates were in the acceptable range of less than 0.1^14^ (0.035 for log OR corresponding to TNF inhibitor in combination with methotrexate therapy compared with TNF inhibitor monotherapy; 0.002 for log OR corresponding to methotrexate monotherapy compared with TNF inhibitor monotherapy; 0.072 for log OR corresponding to Jak inhibitor monotherapy compared with TNF inhibitor monotherapy), suggesting that reporting bias was minimal in the GRA registry (Table 4).

**Table 4.** Sensitivity Analysis of the Extent of Potential Reporting Bias Based on Data From the GRA Registry

Exposure treatment regimen^a^	Estimates from GRA-specific analysis				Standardized difference^d^	Regulatory agreement^e^	Estimate agreement^f^
	Original		Reweighted^b^				
	OR (95% CI)^c^	*P* value	OR (95% CI)^c^	*P* value			
TNF inhibitor monotherapy	1 [Reference]	NA	1 [Reference]	NA	NA	NA	NA
TNF inhibitor plus methotrexate	1.20 (0.80-1.79)	.38	0.96 (0.57-1.64)	.89	0.035	Yes	Yes
Methotrexate monotherapy	2.21 (1.59-3.08)	<.001	2.20 (1.82-2.65)	<.001	0.002	Yes	Yes
Jak inhibitor monotherapy	2.41 (1.46-3.99)	.001	1.88 (1.44-2.45)	<.001	0.072	Yes	Yes

Abbreviations: GRA, COVID-19 Global Rheumatology Alliance; Jak, Janus kinase; NA, not applicable; OR, odds ratio; TNF, tumor necrosis factor.

^a^ The number of patients receiving azathioprine/6-mecaptopurine monotherapy or TNF inhibitors in combination with azathioprine/6-mecaptopurine therapy was too small in the rheumatology clinics to derive estimates for these exposure treatment regimens.

^b^ Estimates were obtained after reweighting the covariate distribution of patients in the GRA registry to match those of rheumatology clinics from health care systems that systematically reported all confirmed and suspected COVID-19 patients, using the inverse odds of sampling weights technique.

^c^ All ORs were derived using hierarchical multivariable mixed-effects logistic regression analysis with calendar month random effects nested within country and adjusted for the following: age, sex, current smoking, immune-mediated disease diagnosis, immune-mediated disease activity (remission vs active), important comorbidities (cardiovascular disease, diabetes, hypertension, obstructive lung disease, interstitial or other chronic lung disease, kidney disease, obesity [body mass index ≥30; calculated as weight in kilograms divided by height in meters squared], and cancer), and receipt of sulfasalazine, hydroxychloroquine or chloroquine, leflunomide, and prednisone-equivalent glucocorticoid dose.

^d^ Standardized difference measured the extent of the difference between the original (potentially biased) and reweighted estimates. Standardized differences were derived from log ORs according to the methods in Franklin et al.^13^ Values <0.1 were considered acceptable standardized differences.^14^

^e^ Regulatory agreement indicates whether original estimates replicated the statistical significance and direction (when estimates were statistically significant) of reweighted estimates.

^f^ Estimate agreement indicates whether the original estimate was within the 95% CI of the reweighted estimates.

A total of 864 patients (14.2%; 112 patients [37.3%] from the PsoProtect registry, 752 patients [21.9%] from the GRA registry, and 0 patients from the SECURE-IBD registry) received a COVID-19 diagnosis based on symptoms alone. Our pooled results remained consistent in a sensitivity analysis that excluded these patients (Figure).

## Discussion

This cohort study found that TNF inhibitor monotherapy was associated with a lower risk of COVID-19–associated hospitalization or death among patients with IMIDs compared with other commonly used treatment regimens, including methotrexate, azathioprine/6-mercaptopurine, and Jak inhibitors. After controlling for active disease and common comorbidities, the odds of hospitalization or death among those who received TNF inhibitor combination therapies vs TNF inhibitor monotherapy depended on the type of additional medication used in the combination regimen. Patients receiving TNF inhibitors in combination with azathioprine/6-mercaptopurine therapy had higher odds of hospitalization or death compared with those receiving TNF inhibitor monotherapy, whereas individuals receiving TNF inhibitors in combination with methotrexate therapy had similar odds of hospitalization or death compared with those receiving TNF inhibitors alone.

The lower odds of unfavorable COVID-19 outcomes among patients receiving TNF inhibitors before SARS-CoV-2 infection has several possible explanations. Although the exact mechanism of SARS-CoV-2–associated hyperinflammation remains uncertain, high serum TNF concentrations at the time of COVID-19 admission have been associated with organ damage and worse COVID-19 outcomes.^15^ Therefore, blocking TNF could inhibit this detrimental immune response. Multiple case series reporting favorable outcomes among patients receiving TNF inhibitor therapy support this assertion.^1,16,17^ Upcoming results from clinical trials investigating the use of TNF inhibitors will enable further evaluation of the association between TNF inhibitor therapy and COVID-19 outcomes.^18,19^

Other possible explanations for our findings include the consequences of non-TNF inhibitor immunosuppressive medications for COVID-19 outcomes. Thiopurine medications are associated with a higher risk of opportunistic viral infections.^20,21,22^ A study examining data from a large registry of patients with IBD found that the receipt of thiopurines, including azathioprine and 6-mercaptopurine, was associated with a higher risk of serious viral infection, specifically infection from species of the Herpesviridae.^23^ Although data regarding other viruses cannot be directly extrapolated to COVID-19, this higher risk highlights the potential for an association between thiopurine use and an increased risk of unfavorable outcomes after SARS-CoV-2 infection. Moreover, a recent study of data from the SECURE-IBD registry reported that thiopurine monotherapy and thiopurines in combination with TNF inhibitor therapy were associated with worse COVID-19 outcomes compared with TNF inhibitor monotherapy.^24^ In contrast, researchers have postulated that methotrexate therapy may decrease the cytokine storm associated with COVID-19.^25,26^ However, our results suggest worse outcomes associated with methotrexate monotherapy compared with TNF inhibitor monotherapy. This association could mean that TNF inhibitor therapy is exerting a protective benefit or that methotrexate therapy is exerting a harmful consequence. Notably, the direction of association was the same for methotrexate used in combination with TNF inhibitors, although the effect estimate crossed the line of no effect, which is possibly associated with the use of lower methotrexate doses in combination therapy compared with monotherapy.^27,28^

The timing of treatment initiation with Jak inhibitors may be an important factor associated with COVID-19 outcomes. The second iteration of the Adaptive COVID-19 Treatment Trial (ACTT-2) suggested a protective effect of treatment with baricitinib in combination with remdesivir therapy against unfavorable COVID-19 outcomes among some subgroups of patients with confirmed severe COVID-19.^29^ However, population-based data from patients receiving Jak inhibitors before COVID-19 diagnosis suggest worse outcomes, which is consistent with the known association between this class of medications and reductions in the innate immune response, producing impaired viral clearance.^30^ In our comparative analyses, we found that Jak inhibitor monotherapy was associated with higher odds of hospitalization or death than TNF inhibitor monotherapy.

### Strengths and Limitations

This study has strengths. These strengths include the robust worldwide collaboration between 3 international registries, which enabled evaluation of a large geographically diverse sample of adults with IMIDs. To our knowledge, this study is the first to pool data across registries to evaluate COVID-19 outcomes among patients with IMIDs. Pooling data increased the power of the study, allowed for more granular analyses of medications, and improved generalizability across IMIDs. Notably, our analyses controlled for active disease, which is only possible through the use of registry data because this variable is not typically available in administrative databases or electronic health records. Furthermore, clinicians or trained staff reported directly to each registry, which likely increased the accuracy of the information.

This study also has limitations. These limitations include the risk of reporting bias because the registries used convenience sampling. However, the results of our sensitivity analysis suggest that reporting bias was not a substantial concern in the GRA registry. The threshold for hospitalization and the ways in which patients are treated for COVID-19 differs over time and across regions. Such differences have the potential to introduce bias if insufficiently accounted for in the analyses. Although we attempted to account for associations in hospitalization or death owing to unmeasured temporal and geographical factors, residual confounding may remain. Additional factors that we were unable to account for included duration and previous lines of IMID therapy. Furthermore, the lack of a global COVID-19 registration system limited the feasibility of including a control group. Although the case report forms were similar, the data domains across registries were not entirely uniform. For example, time and type of COVID-19 diagnosis, rheumatic disease activity, and certain comorbidities were recorded slightly differently across registries. These inconsistencies were addressed, to the extent possible, through the incorporation of registry-level random effects in multilevel modeling and through sensitivity analyses.

## Conclusions

The results of this cohort study suggest that, among patients with IMIDs, receipt of TNF inhibitor monotherapy may be associated with a lower risk of COVID-19–associated hospitalization or death compared with other immunomodulatory treatment regimens. These findings support the continued use of TNF inhibitor monotherapy during the pandemic and warrant further research investigating the association of other biologic therapies with COVID-19 outcomes. Treatment with TNF inhibitor combination therapy was associated with a more favorable safety profile when methotrexate rather than azathioprine/6-mercaptopurine was used, suggesting that clinicians would benefit from weighing the risks vs benefits of deescalating treatment or changing medications when a patient is receiving concomitant TNF inhibitors and azathioprine/6-mercaptopurine.

## Notes

Supplement 1. **eTable.** Adjusted Registry-Specific Odds of COVID-19–Associated Hospitalization or Death

Supplement 2. **Nonauthor Collaborators.** Members of the Psoriasis Patient Registry for Outcomes, Therapy and Epidemiology of COVID-19 Infection (PsoProtect); the Secure Epidemiology of Coronavirus Under Research Exclusion for Inflammatory Bowel Disease (SECURE-IBD); and the COVID-19 Global Rheumatology Alliance (GRA)
