# Supplementary material for: Association Between Tumor Necrosis Factor Inhibitors and the Risk of Hospitalization or Death Among Patients With Immune-Mediated Inflammatory Disease and COVID-19
Source: JAMA Netw Open. 2021 Oct 18;4(10):e2129639. doi: 10.1001/jamanetworkopen.2021.29639 (PMC8524310; doi:10.1001/jamanetworkopen.2021.29639)
Supplement: Supplement 1. — eTable. Adjusted Registry-Specific Odds of COVID-19–Associated Hospitalization or Death [file jamanetwopen-e2129639-s001.pdf]

Supplementary Online Content

Izadi Z, Brenner EJ, Mahil SK, et al. Association between tumor necrosis factor inhibitors and the risk of hospitalization or death among patients with immune-mediated inflammatory disease and COVID-19. *JAMA Netw Open*. 2021;4(10):e2129639. doi:10.1001/jamanetworkopen.2021.29639

eTable. Adjusted Registry-Specific Odds of COVID-19–Associated Hospitalization or Death

This supplementary material has been provided by the authors to give readers additional information about their work.

© 2021 Izadi Z et al. *JAMA Network Open*.

eTable. Adjusted Registry-Specific Odds of COVID-19-Associated Hospitalization or Death.

	GRA N = 3,441		SECURE-IBD N = 2,336		PsoProtect N = 300	
	**OR (95% CI)**	**P**	**OR (95% CI)**	**P**	**OR (95% CI)**	**P**
**Exposure regimens***						
TNFi monotherapy (Ref)	1	--	1	--	1	--
TNFi + Methotrexate	1.20 (0.80-1.79)	0.375	1.59 (0.76-3.34)	0.222		
TNFi + Azathioprine/6MP			1.51 (0.98-2.31)	0.061		
Methotrexate monotherapy	2.21 (1.59-3.08)	<0.001	2.66 (0.95-7.43)	0.062	8.76 (2.94-26.06)	<0.001
Azathioprine/6MP monotherapy	5.28 (1.51-18.43)	0.009	1.50 (1.00-2.24)	0.048		
JAKi monotherapy	2.41 (1.46-3.99)	0.001	0.60 (0.22-1.64)	0.322		
**Diagnoses***						
Rheumatoid arthritis only (Ref)	1	--				
Spondyloarthritis only	1.32 (0.85-2.08)	0.220				
Psoriatic arthritis only	0.89 (0.61-1.28)	0.518				
Other IA or >1 type of IA	0.71 (0.35-1.42)	0.326				
Crohn's disease (Ref)			1	--		
Ulcerative colitis			0.86 (0.24-3.01)	0.808		
IBD unspecified			1.17 (0.83-1.65)	0.377		
**Disease activity**						
Active disease	1.02 (0.78-1.33)	0.886	2.02 (1.45-2.80)	<0.001	0.86 (0.25-2.91)	0.806
**Concomitant medications**						
Sulfasalazine	1.55 (1.03-2.35)	0.037	1.69 (0.72-4.01)	0.231		
Leflunomide	1.97 (1.22-3.18)	0.005				
Hydroxychloroquine or chloroquine	0.93 (0.64-1.34)	0.684				
Mesalamine			1.24 (0.82-1.89)	0.310		
Oral budesonide			2.71 (1.11-0.60)	0.028		
Daily prednisone-equivalent GC dose (per mg)	1.07 (1.03-1.10)	<0.001	1.06 (1.04-1.08)	<0.001		
**Demographics**						
Female	0.79 (0.60-1.04)	0.091	0.82 (0.61-1.11)	0.209	0.37 (0.13-1.11)	0.076
Age (per year)	1.05 (1.04-1.06)	<0.001	1.04 (1.03-1.05)	<0.001	1.04 (1.00-1.09)	0.068
Current smoker	0.77 (0.44-1.36)	0.364	0.92 (0.45-1.87)	0.815	0.49 (0.10-2.39)	0.375
Obesity (BMI ≥30 kg/m^2)	1.75 (1.28-2.38)	<0.001	1.27 (0.85-1.90)	0.240	0.73 (0.23-2.33)	0.598
**Comorbidities**						
Interstitial lung disease	1.61 (0.92-2.81)	0.096	1.58 (0.51-4.89)	0.429		
Obstructive lung disease	2.47 (1.67-3.67)	<0.001	2.34 (1.22-4.48)	0.011	1.05 (0.11-10.02)	0.968
Cardiovascular disease	1.25 (0.81-1.93)	0.316	2.87 (1.55-5.32)	0.001	1.58 (0.34-7.37)	0.564
Diabetes	1.63 (1.17-2.29)	0.004	1.10 (0.55-2.19)	0.785	2.27 (0.75-6.82)	0.146
Hypertension	1.09 (0.83-1.44)	0.516	1.50 (0.92-2.43)	0.102	3.08 (1.01-9.45)	0.049
Kidney disease	3.92 (1.82-8.44)	<0.001	1.76 (0.54-5.76)	0.350		
Cancer	1.32 (0.67-2.59)	0.424	0.77 (0.17-3.41)	0.728	0.85 (0.06-12.85)	0.908

* Categories are mutually exclusive. Estimates obtained using hierarchical multivariable mixed-effects logistic regression with calendar-month random effects nested within country. Models adjusted for all variables shown. Abbreviations: GRA: the Global Rheumatology Alliance registry; SECURE-IBD: the Secure Epidemiology of Coronavirus Under Research Exclusion for Inflammatory Bowel Disease

© 2021 Izadi Z et al. *JAMA Network Open*.

registry; PsoProtect: the Psoriasis Patient Registry for Outcomes, Therapy and Epidemiology of COVID-19 Infection; JAKi: janus kinase inhibitors; MTX: methotrexate; AZA: azathioprine; 6MP: 6-mercaptopurine; TNFi: tumor necrosis factor inhibitor; IA: inflammatory arthritis; IBD: inflammatory bowel disease, GC: glucocorticoid.

© 2021 Izadi Z et al. *JAMA Network Open*.
