# Supplementary material for: Association Between Tumor Necrosis Factor Inhibitors and the Risk of Hospitalization or Death Among Patients With Immune-Mediated Inflammatory Disease and COVID-19
Source: JAMA Netw Open. 2021 Oct 18;4(10):e2129639. doi: 10.1001/jamanetworkopen.2021.29639 (PMC8524310; doi:10.1001/jamanetworkopen.2021.29639)
Supplement: Supplement 2. — Nonauthor Collaborators. Members of the Psoriasis Patient Registry for Outcomes, Therapy and Epidemiology of COVID-19 Infection (PsoProtect); the Secure Epidemiology of Coronavirus Under Research Exclusion for Inflammatory Bowel Disease (SECURE-IBD); and the COVID-19 Global Rheumatology Alliance (GRA) [file jamanetwopen-e2129639-s002.pdf]

Supplemental Online Content: Nonauthor Collaborators
*Indicates required information. Only first name, last name, and suffix will appear in PubMed.

Supplementary Online Material: Nonauthor Collaborators

Group Names: Psoriasis Patient Registry for Outcomes, Therapy and Epidemiology of COVID-19 Infection (PsoProtect), Secure Epidemiology of Coronavirus Under Research Exclusion for Inflammatory Bowel Disease (SECURE-IBD), and COVID-19 Global Rheumatology Alliance (GRA)

***First Name and Middle Initial(s)**	***Last Name**	***Suffix (eg, Jr, III)**	Academic Degrees	Institution	Location (city, state/province, country)	Role or Contribution, eg, chair, principal investigator	Group (if more than 1 Group listed in the byline) and/or Subgroup (eg, Steering Committee)
Hervé	Bachelez		MD, PhD	1. Department of Dermatology, AP-HP Hôpital Saint-Louis<br>2. INSERM U1163, Imagine Institute for Human Genetic Diseases, Université de Paris	Paris, France		PsoProtect, International Scientific Advisory Board
Francesca	Capon		PhD	Department of Medical and Molecular Genetics, School of Basic and Medical Biosciences, Faculty of Life Sciences and Medicine, King's College London	London, United Kingdom		PsoProtect, Study Group
Bola	Coker		MSc	National Institute for Health Research Biomedical Research Centre at Guy's and St Thomas' NHS Foundation Trust	London, United Kingdom		PsoProtect, Study Group
Claudia	De La Cruz		MD	Clinica Dermacross	Santiago, Chile		PsoProtect, International Scientific Advisory Board
Kayleigh J	Mason		PhD	Dermatology Centre, Salford Royal NHS Foundation Trust, The University of Manchester, Manchester Academic Health Science Centre, National Institute for Health Research Manchester Biomedical Research Centre	Manchester, United Kingdom		PsoProtect, Study Group
Paola	Di Meglio		PhD	St. John's Institute of Dermatology, School of Basic & Medical Biosciences, Faculty of Life Sciences & Medicine, King's College London	London, United Kingdom		PsoProtect, Study Group
Joel M	Gelfand		MD, MSCE	1. Department of Dermatology, University of Pennsylvania Perelman School of Medicine<br>2. Department of Biostatistics, Epidemiology and Informatics, Perelman School of Medicine at the University of Pennsylvania	Philadelphia, Pennsylvania, United States of America		PsoProtect, International Scientific Advisory Board
Paolo	Gisondi		MD	Section of Dermatology and Venereology, University of Verona	Verona, Italy		PsoProtect, Steering Committee

Supplemental Online Content: Nonauthor Collaborators

*Indicates required information. Only first name, last name, and suffix will appear in PubMed.

*First Name and Middle Initial(s)	*Last Name	*Suffix (eg, Jr, III)	Academic Degrees	Institution	Location (city, state/province, country)	Role or Contribution, eg, chair, principal investigator	Group (if more than 1 Group listed in the byline) and/or Subgroup (eg, Steering Committee)
Lars	Iversen		MD, PhD	Department of Dermatology, Aarhus University Hospital	Aarhus, Denmark		PsoProtect, International Scientific Advisory Board
Denis	Jullien		MD, PhD	1. Groupe de recherche sur le psoriasis (GrPso) de la Société Française de Dermatologie<br>2. Department of Dermatology, Edouard Herriot Hospital, Hospices Civils de Lyon	1. Paris, France<br>2. Lyon, France		PsoProtect, Steering Committee
Jo	Lambert		MD, PhD	Department of Dermatology, Ghent University	Ghent, Belgium		PsoProtect, Study Group
Sinéad M	Langan		PhD, FRCP	St John's Institute of Dermatology, Guy's and St Thomas' NHS Foundation Trust and King's College London	London, United Kingdom		PsoProtect, Steering Committee
Helen	McAteer		BSc	The Psoriasis Association	Northampton, United Kingdom		PsoProtect, Steering Committee
Freya	Meynell		MSc	St John's Institute of Dermatology, Guy's and St Thomas' NHS Foundation Trust and King's College London	London, United Kingdom		PsoProtect, Study Group
Lucy	Moorhead		MA	St John's Institute of Dermatology, Guy's and St Thomas' NHS Foundation Trust and King's College London	London, United Kingdom		PsoProtect, Study Group
Luigi	Naldi		MD	Centro Studi GISED	Bergamo, Italy		PsoProtect, International Scientific Advisory Board
Luis	Puig		MD, PhD	Department of Dermatology, Hospital de la Santa Creu i Sant Pau, Universitat Autònoma de Barcelona	Barcelona, Spain		PsoProtect, Steering Committee
Nick J	Reynolds		MD, FRCP	1. Department of Dermatology and NIHR Newcastle Biomedical Research Centre, Newcastle Hospitals NHS Foundation Trust<br>2. Translational and Clinical Research Institute, Newcastle University	Newcastle upon Tyne, United Kingdom		PsoProtect, International Scientific Advisory Board
Phyllis	Spuls		MD, PhD	Department of Dermatology, Amsterdam Public Health/Infection and Immunology, Amsterdam University Medical Centers	Amsterdam, The Netherlands		PsoProtect, International Scientific Advisory Board

Supplemental Online Content: Nonauthor Collaborators

*Indicates required information. Only first name, last name, and suffix will appear in PubMed.

*First Name and Middle Initial(s)	*Last Name	*Suffix (eg, Jr, III)	Academic Degrees	Institution	Location (city, state/province, country)	Role or Contribution, eg, chair, principal investigator	Group (if more than 1 Group listed in the byline) and/or Subgroup (eg, Steering Committee)
Tiago	Torres		MD, PhD	Department of Dermatology Centro Hospitalar Universitário do Porto	Porto, Portugal		PsoProtect, Study Group
Teresa	Tsakok		MRCP	St John’s Institute of Dermatology, Guy’s and St Thomas’ NHS Foundation Trust and King's College London	London, United Kingdom		PsoProtect, Study Group
Alexandra	Vincent			National Institute for Health Research Biomedical Research Centre at Guy’s and St Thomas’ NHS Foundation Trust	London, United Kingdom		PsoProtect, Study Group
Richard B	Warren		PhD, FRCP	Dermatology Centre, Salford Royal NHS Foundation Trust, The University of Manchester, Manchester Academic Health Science Centre, National Institute for Health Research Manchester Biomedical Research Centre	Manchester, United Kingdom		PsoProtect, International Scientific Advisory Board
Hoseah	Waweru		MD	The International Federation of Psoriasis Associations (IFPA)	Bromma, Sweden		PsoProtect, Steering Committee
Siew	Ng		MBBS, PhD	Department of Medicine and Therapeutics, Institute of Di	Shatin, NT, Hong Kong SAR,	Served as a SECURE-IBD advisor. Helped to raise awareness of the SECURE-IBD database and encourage providers to report cases to the database.	SECURE-IBD, International Advisory Committee
Richard	Gearry		MBChB	University of Otago Department of Medicine	Christchurch Central City, C	Served as a SECURE-IBD advisor. Helped to raise awareness of the SECURE-IBD database and encourage providers to report cases to the database.	SECURE-IBD, International Advisory Committee

Supplemental Online Content: Nonauthor Collaborators

*Indicates required information. Only first name, last name, and suffix will appear in PubMed.

*First Name and Middle Initial(s)	*Last Name	*Suffix (eg, Jr, III)	Academic Degrees	Institution	Location (city, state/province, country)	Role or Contribution, eg, chair, principal investigator	Group (if more than 1 Group listed in the byline) and/or Subgroup (eg, Steering Committee)
Walter	Reinisch		MD	Medical University of Vienna	Klinische Abt. Gastroentero	Served as a SECURE-IBD advisor. Helped to raise awareness of the SECURE-IBD database and encourage providers to report cases to the database.	SECURE-IBD, International Advisory Committee
Jean-Francois	Rahier		MD, PhD	Université catholique de Louvain, CHU UCL Namur	Place de l'Université, Ottigr	Served as a SECURE-IBD advisor. Helped to raise awareness of the SECURE-IBD database and encourage providers to report cases to the database.	SECURE-IBD, International Advisory Committee
James	Lewis		MD, MSCE	The University of Pennsylvania	Philadelphia, Pennsylvania,	Served as a SECURE-IBD advisor. Helped to raise awareness of the SECURE-IBD database and encourage providers to report cases to the database.	SECURE-IBD, International Advisory Committee

Supplemental Online Content: Nonauthor Collaborators

*Indicates required information. Only first name, last name, and suffix will appear in PubMed.

***First Name and Middle Initial(s)**	***Last Name**	***Suffix (eg, Jr, III)**	Academic Degrees	Institution	Location (city, state/province, country)	Role or Contribution, eg, chair, principal investigator	Group (if more than 1 Group listed in the byline) and/or Subgroup (eg, Steering Committee)
Gilaad	Kaplan		MD, MPH,	University of Calgary, Departments of Medicine and Com	Calgary, Alberta, T2N 4Z6, C	Served as a SECURE-IBD advisor. Helped to raise awareness of the SECURE-IBD database and encourage providers to report cases to the database.	SECURE-IBD, International Advisory Committee
Flavio	Steinwurz		MD, MSc, M	Hospital Israelita Albert Einstein	Jardim Leonor, São Paulo, B	Served as a SECURE-IBD advisor. Helped to raise awareness of the SECURE-IBD database and encourage providers to report cases to the database.	SECURE-IBD, International Advisory Committee
Michele	Kissous-Hunt		PA-C, DFAA	Mount Sinai Medical Center	New York, New York, United States	Served as a SECURE-IBD advisor. Helped to raise awareness of the SECURE-IBD database and encourage providers to report cases to the database.	SECURE-IBD, International Advisory Committee

Supplemental Online Content: Nonauthor Collaborators

*Indicates required information. Only first name, last name, and suffix will appear in PubMed.

***First Name and Middle Initial(s)**	***Last Name**	***Suffix (eg, Jr, III)**	Academic Degrees	Institution	Location (city, state/province, country)	Role or Contribution, eg, chair, principal investigator	Group (if more than 1 Group listed in the byline) and/or Subgroup (eg, Steering Committee)
Irene	Modesto		MD, PhD	Pfizer Inc.	New York, New York, Unite	Served as a SECURE-IBD advisor. Helped to raise awareness of the SECURE-IBD database and encourage providers to report cases to the database.	SECURE-IBD, International Advisory Committee
Marishka	Konings			International Organization for the Study of Inflammatory Bowel Disease	BAARN, The Netherlands	Served as a SECURE-IBD advisor. Helped to raise awareness of the SECURE-IBD database and encourage providers to report cases to the database.	SECURE-IBD, International Advisory Committee
Brahim	Dahou			Association Rhumatologues Algériens Privés (ARAP)	Algiers, Algeria	Collaborator	GRA
Gimena	Gómez			Argentine Society of Rheumatology	Buenos Aires, Argentina	Collaborator	GRA
Karen	Roberts			Argentine Society of Rheumatology	Buenos Aires, Argentina	Collaborator	GRA
Roberto M	Baez			Argentine Society of Rheumatology	Buenos Aires, Argentina	Collaborator	GRA
Vanessa V	Castro Coello			Argentine Society of Rheumatology	Buenos Aires, Argentina	Collaborator	GRA
María J	Haye Salinas			Argentine Society of Rheumatology	Buenos Aires, Argentina	Collaborator	GRA
Federico N	Maldonado			Argentine Society of Rheumatology	Buenos Aires, Argentina	Collaborator	GRA
Alvaro A	Reyes			Argentine Society of Rheumatology	Buenos Aires, Argentina	Collaborator	GRA
Gelsomina	Alle			Argentine Society of Rheumatology	Buenos Aires, Argentina	Collaborator	GRA
Romina	Tanten			Argentine Society of Rheumatology	Buenos Aires, Argentina	Collaborator	GRA
Hernán	Maldonado Ficco			Argentine Society of Rheumatology	Buenos Aires, Argentina	Collaborator	GRA
Romina	Nieto			Argentine Society of Rheumatology	Buenos Aires, Argentina	Collaborator	GRA
Carla	Gobbi			Argentine Society of Rheumatology	Buenos Aires, Argentina	Collaborator	GRA
Yohana	Tissera			Argentine Society of Rheumatology	Buenos Aires, Argentina	Collaborator	GRA
Cecilia	Pisoni			Argentine Society of Rheumatology	Buenos Aires, Argentina	Collaborator	GRA
Alba	Paula			Argentine Society of Rheumatology	Buenos Aires, Argentina	Collaborator	GRA
Juan A	Albiero			Argentine Society of Rheumatology	Buenos Aires, Argentina	Collaborator	GRA

Supplemental Online Content: Nonauthor Collaborators

*Indicates required information. Only first name, last name, and suffix will appear in PubMed.

*First Name and Middle Initial(s)	*Last Name	*Suffix (eg, Jr, III)	Academic Degrees	Institution	Location (city, state/province, country)	Role or Contribution, eg, chair, principal investigator	Group (if more than 1 Group listed in the byline) and/or Subgroup (eg, Steering Committee)
Maria M	Schmid			Argentine Society of Rheumatology	Buenos Aires, Argentina	Collaborator	GRA
Micaela	Cosatti			Argentine Society of Rheumatology	Buenos Aires, Argentina	Collaborator	GRA
Maria J	Gamba			Argentine Society of Rheumatology	Buenos Aires, Argentina	Collaborator	GRA
Carlevaris	Leandro			Argentine Society of Rheumatology	Buenos Aires, Argentina	Collaborator	GRA
María A	Cusa			Argentine Society of Rheumatology	Buenos Aires, Argentina	Collaborator	GRA
Noelia	German			Argentine Society of Rheumatology	Buenos Aires, Argentina	Collaborator	GRA
Veronica	Bellomio			Argentine Society of Rheumatology	Buenos Aires, Argentina	Collaborator	GRA
Lorena	Takashima			Argentine Society of Rheumatology	Buenos Aires, Argentina	Collaborator	GRA
Mariana	Pera			Argentine Society of Rheumatology	Buenos Aires, Argentina	Collaborator	GRA
Karina	Cogo			Argentine Society of Rheumatology	Buenos Aires, Argentina	Collaborator	GRA
Maria S	Gálvez Elkin			Argentine Society of Rheumatology	Buenos Aires, Argentina	Collaborator	GRA
María A	Medina			Argentine Society of Rheumatology	Buenos Aires, Argentina	Collaborator	GRA
Veronica	Savio			Argentine Society of Rheumatology	Buenos Aires, Argentina	Collaborator	GRA
Romina	Rojas Tessel			Argentine Society of Rheumatology	Buenos Aires, Argentina	Collaborator	GRA
Rodolfo P	Alamino			Argentine Society of Rheumatology	Buenos Aires, Argentina	Collaborator	GRA
Marina L	Werner			Argentine Society of Rheumatology	Buenos Aires, Argentina	Collaborator	GRA
Sofía	Ornella			Argentine Society of Rheumatology	Buenos Aires, Argentina	Collaborator	GRA
Luciana	Casalla			Argentine Society of Rheumatology	Buenos Aires, Argentina	Collaborator	GRA
Maria	de la Vega			Argentine Society of Rheumatology	Buenos Aires, Argentina	Collaborator	GRA
María	Severina			Argentine Society of Rheumatology	Buenos Aires, Argentina	Collaborator	GRA
Mercedes	García			Argentine Society of Rheumatology	Buenos Aires, Argentina	Collaborator	GRA
Luciana	Gonzalez Lucero			Argentine Society of Rheumatology	Buenos Aires, Argentina	Collaborator	GRA
Cecilia	Romeo			Argentine Society of Rheumatology	Buenos Aires, Argentina	Collaborator	GRA
Sebastián	Moyano			Argentine Society of Rheumatology	Buenos Aires, Argentina	Collaborator	GRA
Tatiana	Barbich			Argentine Society of Rheumatology	Buenos Aires, Argentina	Collaborator	GRA
Ana	Bertoli			Argentine Society of Rheumatology	Buenos Aires, Argentina	Collaborator	GRA
Andrea	Baños			Argentine Society of Rheumatology	Buenos Aires, Argentina	Collaborator	GRA
Sandra	Petruzzelli			Argentine Society of Rheumatology	Buenos Aires, Argentina	Collaborator	GRA
Carla	Matellan			Argentine Society of Rheumatology	Buenos Aires, Argentina	Collaborator	GRA
Silvana	Conti			Argentine Society of Rheumatology	Buenos Aires, Argentina	Collaborator	GRA
Maria A	Lazaro			Argentine Society of Rheumatology	Buenos Aires, Argentina	Collaborator	GRA
Gustavo F	Rodriguez Gil			Argentine Society of Rheumatology	Buenos Aires, Argentina	Collaborator	GRA
Fabian	Risueño			Argentine Society of Rheumatology	Buenos Aires, Argentina	Collaborator	GRA
Maria I	Quaglia			Argentine Society of Rheumatology	Buenos Aires, Argentina	Collaborator	GRA
Julia	Scafati			Argentine Society of Rheumatology	Buenos Aires, Argentina	Collaborator	GRA
Natalia L	Cuchiaro			Argentine Society of Rheumatology	Buenos Aires, Argentina	Collaborator	GRA
Jonathan E	Rebak			Argentine Society of Rheumatology	Buenos Aires, Argentina	Collaborator	GRA

*Indicates required information. Only first name, last name, and suffix will appear in PubMed.

*First Name and Middle Initial(s)	*Last Name	*Suffix (eg, Jr, III)	Academic Degrees	Institution	Location (city, state/province, country)	Role or Contribution, eg, chair, principal investigator	Group (if more than 1 Group listed in the byline) and/or Subgroup (eg, Steering Committee)
Susana I	Pineda			Argentine Society of Rheumatology	Buenos Aires, Argentina	Collaborator	GRA
María E	Calvo			Argentine Society of Rheumatology	Buenos Aires, Argentina	Collaborator	GRA
Eugenia	Picco			Argentine Society of Rheumatology	Buenos Aires, Argentina	Collaborator	GRA
Josefina G	Yanzi			Argentine Society of Rheumatology	Buenos Aires, Argentina	Collaborator	GRA
Pablo	Maid			Argentine Society of Rheumatology	Buenos Aires, Argentina	Collaborator	GRA
Debora	Guaglianone			Argentine Society of Rheumatology	Buenos Aires, Argentina	Collaborator	GRA
Julieta S	Morbiducci			Argentine Society of Rheumatology	Buenos Aires, Argentina	Collaborator	GRA
Sabrina	Porta			Argentine Society of Rheumatology	Buenos Aires, Argentina	Collaborator	GRA
Natalia	Herscovich			Argentine Society of Rheumatology	Buenos Aires, Argentina	Collaborator	GRA
José L	Velasco Zamora			Argentine Society of Rheumatology	Buenos Aires, Argentina	Collaborator	GRA
Boris	Kisluk			Argentine Society of Rheumatology	Buenos Aires, Argentina	Collaborator	GRA
Maria S	Castaños Menescardi			Argentine Society of Rheumatology	Buenos Aires, Argentina	Collaborator	GRA
Rosana	Gallo			Argentine Society of Rheumatology	Buenos Aires, Argentina	Collaborator	GRA
María V	Martire			Argentine Society of Rheumatology	Buenos Aires, Argentina	Collaborator	GRA
Carla	Maldini			Argentine Society of Rheumatology	Buenos Aires, Argentina	Collaborator	GRA
Cecilia	Goizueta			Argentine Society of Rheumatology	Buenos Aires, Argentina	Collaborator	GRA
Sabrina S	de la Vega Fernandez			Argentine Society of Rheumatology	Buenos Aires, Argentina	Collaborator	GRA
Carolina	Aeschlimann			Argentine Society of Rheumatology	Buenos Aires, Argentina	Collaborator	GRA
Gisela	Subils			Argentine Society of Rheumatology	Buenos Aires, Argentina	Collaborator	GRA
Eva	Rath			Hanusch Krankenhaus, Vienna	Vienna, Austria	Collaborator	GRA
Yves	Piette			AZ Sint-Jan Brugge	Bruges, Belgium	Collaborator	GRA
Mieke	Devinck			AZ Sint-Lucas Brugge	Bruges, Belgium	Collaborator	GRA
Bea	Maeyaert			AZ Sint-Lucas Brugge	Bruges, Belgium	Collaborator	GRA
Francinne	Machado Ribeiro			Hospital Universitário Pedro Ernesto Universidade do Esta	Rio de Janeiro, Brazil	Collaborator	GRA
Sandra L	Euzebio Ribeiro			Federal University of Amazonas	Manaus, Brazil	Collaborator	GRA
Marcelo	Pinheiro			Universidade Federal De São Paulo Escola Paulista de Me	São Paulo, Brazil	Collaborator	GRA
Sebastián	Ibáñez			Clínica Alemana de Santiago	Santiago, Chile	Collaborator	GRA
Anne-Marie	Chassin Trubert			Complejo Hospitalario San José	San José, Chile	Collaborator	GRA
Lingli	Dong			Tongji Hospital	Wuhan, China	Collaborator	GRA
Lui	Cajas			Clinica Universitaria Colombia - Centro Medico Providenc	Bogotá, Colombia	Collaborator	GRA
Marko	Barešić			University Hospital Center Zagreb	Zagreb, Croatia	Collaborator	GRA
Branimir	Anić			Div Clin Immunol Rheumatol; Dept Int Med, School of Me	Zagreb, Croatia	Collaborator	GRA
Melanie-Ivana	Čulo			University Hospital Dubrava, Zagreb	Zagreb, Croatia	Collaborator	GRA
Tea A	Pavelić			Clinical Hospital Center Rijeka	Rijeka, Croatia	Collaborator	GRA
Kristina K	Stranski			University hospital Osijek	Osijek, Croatia	Collaborator	GRA
Boris	Karanovic			UHC Zagreb	Zagreb, Croatia	Collaborator	GRA
Jiri	Vencovsky			Institute of Rheumatology, Prague	Prague, Czechia	Collaborator	GRA

*Indicates required information. Only first name, last name, and suffix will appear in PubMed.

*First Name and Middle Initial(s)	*Last Name	*Suffix (eg, Jr, III)	Academic Degrees	Institution	Location (city, state/province, country)	Role or Contribution, eg, chair, principal investigator	Group (if more than 1 Group listed in the byline) and/or Subgroup (eg, Steering Committee)
Marta	Píchová			Medipont plus s.ro. , České Budějovice	České Budějovice, Czechia	Collaborator	GRA
Maria	Filkova			Institute of Rheumatology, Prague	Prague, Czechia	Collaborator	GRA
Hesham	Hamoud			Al Azhar University Hospitals	Nasr City, Egypt	Collaborator	GRA
Dimitrios	Vassilopoulos			Hippokration General Hospital, Athens	Athens, Greece	Collaborator	GRA
Gabriela M	Guzman Melgar			Hospital del Valle, Honduras	San Pedro Sula, Honduras	Collaborator	GRA
Ho	So			Chinese University of Hong Kong	Shatin, Hong Kong	Collaborator	GRA
Márta	Király			Petz Aladár University Teaching Hospital, Győr	Győr, Hungary	Collaborator	GRA
Mahdi	Vojdanian			Iran Rheumatology Center	Tehran, Iran	Collaborator	GRA
Alexandra	Balbir Gurman			Rambam Rheumatology Institute, Haifa	Haifa, Israel	Collaborator	GRA
Fatemah	Abutiban			Kuwait Rheumatology Association	Kuwait City, Kuwait	Collaborator	GRA
Julija	Zepa			Pauls Stradins Clinical University Hospital, Riga	Riga, Latvia	Collaborator	GRA
Inita	Bulina			Pauls Stradins Clinical University hospital, Riga	Riga, Latvia	Collaborator	GRA
Loreta	Bukauskiene			Klaipeda university hospital	Klaipėda, Lithuania	Collaborator	GRA
Beatriz E	Zazueta Montiel			Centro Medico del Angel	Cuautla, Mexico	Collaborator	GRA
Angel A	Castillo Ortiz			Centro Medico Las Americas	Tijuana, Mexico	Collaborator	GRA
Erick	Zamora Tehozol			Centro Medico Pensiones	Mérida, Mexico	Collaborator	GRA
David	Vega Morales			Hospital General de Zona #17	Monterrey, Mexico	Collaborator	GRA
Diana	Cervántes Rosete			Instituto Nacional de Ciencias Médicas y Nutrición Salvad	Mexico City, Mexico	Collaborator	GRA
Eduardo	Martín Nares			Instituto Nacional de Ciencias Médicas y Nutrición Salvad	Mexico City, Mexico	Collaborator	GRA
Tatiana S	Rodriguez Reyna			Instituto Nacional de Ciencias Médicas y Nutrición Salvad	Mexico City, Mexico	Collaborator	GRA
Marina	Rull Gabayet			Instituto Nacional de Ciencias Médicas y Nutrición Salvad	Mexico City, Mexico	Collaborator	GRA
Deshiré	Alpízar Rodríguez			Mexican College of Rheumatology	Mexico City, Mexico	Collaborator	GRA
Fedra	Irazoque			Private Practice	Mexico City, Mexico	Collaborator	GRA
Xochitl	Jimenez			Centro Medico Naval	Mexico City, Mexico	Collaborator	GRA
Lenny	Geurts van Bon			Ziekenhuisgroep Twente	Hengelo, Netherlands	Collaborator	GRA
Theo	Zijlstra			Isala Hospital, Zwolle	Zwolle, Netherlands	Collaborator	GRA
Monique	Hoekstra			Isala Hospital, Zwolle	Zwolle, Netherlands	Collaborator	GRA
Nasra	Al Adhoubi			Royal Hospital	Muscat, Oman	Collaborator	GRA
Babur	Salim			Fauji Foundation Hospital	Rawalpindi, Pakistan	Collaborator	GRA
Enrique	Giraldo			Complejo Hospitalario	Panama City, Panama	Collaborator	GRA
Ariel	Salinas			Hospital Essalud Alberto Sabogal Sologuren	Bellavista District, Peru	Collaborator	GRA
Manuel	Ugarte Gil			Universidad Científica del Sur-Hospital Guillermo Almenar	Lima, Peru	Collaborator	GRA
Jarosław	Nowakowski			University Hospital, Krakow	Krakow, Poland	Collaborator	GRA
Richard	Conway			St James' Hospital, Dublin	Dublin, Republic of Ireland	Collaborator	GRA
Rachael	Flood			Tallaght University Hospital	Dublin, Republic of Ireland	Collaborator	GRA
Geraldine	McCarthy			Mater Misericordiae University Hospital	Dublin, Republic of Ireland	Collaborator	GRA
Ioana	Felea			County Emergency Hospital, Cluj Napoca	Cluj-Napoca, Romania	Collaborator	GRA

Supplemental Online Content: Nonauthor Collaborators

*Indicates required information. Only first name, last name, and suffix will appear in PubMed.

*First Name and Middle Initial(s)	*Last Name	*Suffix (eg, Jr, III)	Academic Degrees	Institution	Location (city, state/province, country)	Role or Contribution, eg, chair, principal investigator	Group (if more than 1 Group listed in the byline) and/or Subgroup (eg, Steering Committee)
Ileana	Filipescu			County Emergency Hospital, Cluj Napoca	Cluj-Napoca, Romania	Collaborator	GRA
Simona	Rednic			County Emergency Hospital, Cluj Napoca	Cluj-Napoca, Romania	Collaborator	GRA
Laura	Groseanu			Sf Maria Clinical Hospital, Bucharest	Bucharest, Romania	Collaborator	GRA
Maria M	Tamas			County Emergency Hospital, Cluj Napoca	Cluj-Napoca, Romania	Collaborator	GRA
Vanda	Mlynarikova			National Institute of Rheumatic Diseases, Piešťany	Piešťany, Slovak Republic	Collaborator	GRA
Martina	Skamlova			FNSPFDR, Banská Bystrica	Banská Bystrica, Slovak Rep	Collaborator	GRA
Martin	Zlnay			National Institute of Rheumatic Diseases, Piešťany	Piešťany, Slovak Republic	Collaborator	GRA
Dagmar	Mičeková			National Institute of Rheumatic Diseases, Piešťany	Piešťany, Slovak Republic	Collaborator	GRA
Lubica	Capova			University Hospital, Bratislava	Bratislava, Slovak Republic	Collaborator	GRA
Zelmira	Macejova			University Hospital, Košice	Košice, Slovak Republic	Collaborator	GRA
Emőke	Šteňová			University Hospital, Bratislava	Bratislava, Slovak Republic	Collaborator	GRA
Helena	Raffayova			National Institute of Rheumatic Diseases, Piešťany	Piešťany, Slovak Republic	Collaborator	GRA
Gabriela	Belakova			Medman s.r.o., Martin	Martin, Slovak Republic	Collaborator	GRA
Eva	Strakova			Faculty hospital Prešov	Prešov, Slovak Republic	Collaborator	GRA
Marieta	Senčarová			Louis Pasteur University Hospital, Košice	Košice, Slovak Republic	Collaborator	GRA
Soňa	Žlnayová			Poliklinika MarMedico, s.r.o., Nové Mesto nad Váhom	Nové Mesto nad Váhom, Sl	Collaborator	GRA
Anna	Sabová			súkromná reumatologická ambulancia, Vranov nad Topľo	Vranov nad Topľou, Slovak	Collaborator	GRA
Daniela	Spisakova			University Hospital od L. Pasteur Kosice	Košice, Slovak Republic	Collaborator	GRA
Mária	Oetterová			Safarik University hospital, Kosice	Košice, Slovak Republic	Collaborator	GRA
Olga	Lukacova			National Institute of Rheumatic Diseases, Piešťany	Piešťany, Slovak Republic	Collaborator	GRA
Martina	Bakosova			UNB Nemocnica Stare Mesto, Bratislava	Bratislava, Slovak Republic	Collaborator	GRA
Alojzija	Hocevar			UMC Ljubljana	Ljubljana, Slovenia	Collaborator	GRA
Natalia	de la Torre Rubio			Hospital Universitario Puerta de Hierro Majadahonda	Majadahonda, Spain	Collaborator	GRA
Juan J	Alegre Sancho			Hospital Universitari Dr Peset, Valencia	Valencia, Spain	Collaborator	GRA
Montserrat	Corteguera Coro			Complejo Asistencial Avila	Avila, Spain	Collaborator	GRA
Juan C	Cobeta Garcia			Hospital Ernest Lluch, Calatayud	Calatayud, Spain	Collaborator	GRA
Maria C	Torres Martin			Hospital Nuestra Senora Sonsoles, Avila	Avila, Spain	Collaborator	GRA
Jose	Campos			Hospital Universitario Puerta de Hierro	Majadahonda, Spain	Collaborator	GRA
Jose A	Gomez Puerta			Hospital Clinic Barcelona	Barcelona, Spain	Collaborator	GRA
Gozd K	Yardımcı			Hacettepe University Faculty of Medicine, Ankara	Ankara, Turkey	Collaborator	GRA
Servet	Akar			Izmir Katip Celebi University Atatürk Training and Researc	Izmir, Turkey	Collaborator	GRA
Ozan C	Icacan			Bakırköy Dr. Sadi Konuk Research And Training Hospital, I	Istanbul, Turkey	Collaborator	GRA
Selda	ÇELİK			BAKIRKOY DR SADI KONUK EDUCATIONAL AND RESEARCH	Istanbul, Turkey	Collaborator	GRA
Viktoriia	Vasylets			Multifield Medical Centre, Odessa	Odessa, Ukraine	Collaborator	GRA
Su-Ann	Yeoh			University College London Hospital, London	London, United Kingdom	Collaborator	GRA
Claire	Vandevelde			Leeds Teaching Hospitals NHS Trust	Leeds, United Kingdom	Collaborator	GRA
Sasha	Dunt			Countess of Chester NHS Foundation Trust	Chester, United Kingdom	Collaborator	GRA

Supplemental Online Content: Nonauthor Collaborators
*Indicates required information. Only first name, last name, and suffix will appear in PubMed.

*First Name and Middle Initial(s)	*Last Name	*Suffix (eg, Jr, III)	Academic Degrees	Institution	Location (city, state/province, country)	Role or Contribution, eg, chair, principal investigator	Group (if more than 1 Group listed in the byline) and/or Subgroup (eg, Steering Committee)
Jane	Leeder			Norfolk & Norwich University Hospital	Norfolk, United Kingdom	Collaborator	GRA
Elizabeth	Macphie			Lancashire and South Cumbria NHS Foundation Trust	Preston, United Kingdom	Collaborator	GRA
Rosaria	Salerno			King's College Hospital	London, United Kingdom	Collaborator	GRA
Christine	Graver			Hampshire Hospitals NHS Trust	Basingstoke, United Kingdo	Collaborator	GRA
Katie	Williams			York District Hospital	York, United Kingdom	Collaborator	GRA
Sheila	O'Reilly			Royal Derby Hospital	Derby, United Kingdom	Collaborator	GRA
Kirsty	Devine			York/Scarborough Hospitals	York, United Kingdom	Collaborator	GRA
Jennifer	Tyler			Royal United Hospital, Bath	Bath, United Kingdom	Collaborator	GRA
Elizabeth	Warner			Lister Hospital	London, United Kingdom	Collaborator	GRA
James	Pilcher			University Hospital Lewisham	London, United Kingdom	Collaborator	GRA
Samir	Patel			Queen Elizabeth hospital Woolwich	London, United Kingdom	Collaborator	GRA
Elena	Nikiphorou			King's College Hospital	London, United Kingdom	Collaborator	GRA
Laura	Chadwick			St Helens & Knowsley NHS Foundation Trust	Whiston, United Kingdom	Collaborator	GRA
Caroline M	Jones			Llandudno Hospital	Llandudno, United Kingdom	Collaborator	GRA
Beverley	Harrison			Salford Royal NHS FT	Salford, United Kingdom	Collaborator	GRA
Lucy	Thornton			Bradford Royal Infirmary	Bradford, United Kingdom	Collaborator	GRA
Diana	O'Kane			RNHRD at Royal United Hospital Bath	Bath, United Kingdom	Collaborator	GRA
Lucia	Fusi			King's College Hospital	London, United Kingdom	Collaborator	GRA
Audrey	Low			Salford Royal NHS FT	Salford, United Kingdom	Collaborator	GRA
Sarah	Horton			Minerva Health Centre	Preston, United Kingdom	Collaborator	GRA
Shraddha	Jatwani			Albert Einstein Medical Center, PA	Philadelphia, Pennsylvania,	Collaborator	GRA
Sara	Baig			Arthritis and Rheumatology Consultants, PA	Edina, Minnesota, United S	Collaborator	GRA
Hammad	Bajwa			Arthritis and Rheumatology Consultants, PA	Edina, Minnesota, United S	Collaborator	GRA
Vernon	Berglund			Arthritis and Rheumatology Consultants, PA	Edina, Minnesota, United S	Collaborator	GRA
Angela	Dahle			Arthritis and Rheumatology Consultants, PA	Edina, Minnesota, United S	Collaborator	GRA
Walter	Dorman			Arthritis and Rheumatology Consultants, PA	Edina, Minnesota, United S	Collaborator	GRA
Jody	Hargrove			Arthritis and Rheumatology Consultants, PA	Edina, Minnesota, United S	Collaborator	GRA
Maren	Hilton			Arthritis and Rheumatology Consultants, PA	Edina, Minnesota, United S	Collaborator	GRA
Nicholas	Lebedoff			Arthritis and Rheumatology Consultants, PA	Edina, Minnesota, United S	Collaborator	GRA
Susan	Leonard			Arthritis and Rheumatology Consultants, PA	Edina, Minnesota, United S	Collaborator	GRA
Jennifer	Morgan			Arthritis and Rheumatology Consultants, PA	Edina, Minnesota, United S	Collaborator	GRA
Emily	Pfeifer			Arthritis and Rheumatology Consultants, PA	Edina, Minnesota, United S	Collaborator	GRA
Archibald	Skemp			Arthritis and Rheumatology Consultants, PA	Edina, Minnesota, United S	Collaborator	GRA
Jeffrey	Wilson			Arthritis and Rheumatology Consultants, PA	Edina, Minnesota, United S	Collaborator	GRA
Anne	Wolff			Arthritis and Rheumatology Consultants, PA	Edina, Minnesota, United S	Collaborator	GRA
Eduardo	Cepeda			Austin Diagnostic Clinic	Austin, Texas, United States	Collaborator	GRA
Derrick	Todd			Brigham and Women's Hospital	Boston, Massachusetts, Un	Collaborator	GRA

Supplemental Online Content: Nonauthor Collaborators

*Indicates required information. Only first name, last name, and suffix will appear in PubMed.

***First Name and Middle Initial(s)**	***Last Name**	***Suffix (eg, Jr, III)**	Academic Degrees	Institution	Location (city, state/province, country)	Role or Contribution, eg, chair, principal investigator	Group (if more than 1 Group listed in the byline) and/or Subgroup (eg, Steering Committee)
Denise	Hare			Capital Health Rheumatology	Hopewell Township, New Je	Collaborator	GRA
Cassandra	Calabrese			Cleveland Clinic	Cleveland, Ohio, United Sta	Collaborator	GRA
Christopher	Adams			East Alabama Medical Center	Opelika, Alabama, United S	Collaborator	GRA
Arezou	Khosroshahi			Emory University	Atlanta, Georgia, United Sta	Collaborator	GRA
Adam	Kilian			George Washington University	Washington, D.C., United S	Collaborator	GRA
Douglas	White			Gundersen Health System	La Crosse, Wisconsin, Unite	Collaborator	GRA
Melanie	Winter			Gundersen Health System	La Crosse, Wisconsin, Unite	Collaborator	GRA
Theodore	Fields			Hospital for Special Surgery	New York City, NY, United S	Collaborator	GRA
Caroline	Siegel			Hospital for Special Surgery	New York City, NY, United S	Collaborator	GRA
Nicole	Daver			Institute of Rheumatic and Autoimmune Diseases	Summit, New Jersey, Unite	Collaborator	GRA
Melissa	Harvey			Institute of Rheumatic and Autoimmune Diseases	Summit, New Jersey, Unite	Collaborator	GRA
Neil	Kramer			Institute of Rheumatic and Autoimmune Diseases	Summit, New Jersey, Unite	Collaborator	GRA
Concetta	Lamore			Institute of Rheumatic and Autoimmune Diseases	Summit, New Jersey, Unite	Collaborator	GRA
Suneya	Hogarty			Integrative Arthritis and Pain Consultants	Goldsboro, North Carolina,	Collaborator	GRA
Karen	Yeter			Kaiser Permanente	Oakland, California, United	Collaborator	GRA
Faizah	Siddique			Loyola University Medical Center	Hines, Illinois, United States	Collaborator	GRA
Byung	Ban			Medstar Georgetown University Hospital	Washington, D.C., United S	Collaborator	GRA
Tamar	Tanner			Montefiore Medical Center	Bronx, NY, United States of	Collaborator	GRA
Eric	Ruderman			Northwestern Memorial	Chicago, Illinois, United Sta	Collaborator	GRA
William	Davis			Ochsner Medical Center Rheumatology Department	Jefferson, Louisiana, United	Collaborator	GRA
Robert	Quinet			Ochsner Medical Center Rheumatology Department	Jefferson, Louisiana, United	Collaborator	GRA
Evangeline	Scopelitis			Ochsner Medical Center Rheumatology Department	Jefferson, Louisiana, United	Collaborator	GRA
Karen	Toribio			Ochsner Medical Center Rheumatology Department	Jefferson, Louisiana, United	Collaborator	GRA
Tameka	Webb Detiege			Ochsner Medical Center Rheumatology Department	Jefferson, Louisiana, United	Collaborator	GRA
Jerald	Zakem			Ochsner Medical Center Rheumatology Department	Jefferson, Louisiana, United	Collaborator	GRA
Khurram	Abbass			Private Practice	San Jose, California, United	Collaborator	GRA
Gilbert	Kepecs			Private Practice	Hackensack, New Jersey, U	Collaborator	GRA
Lilliam	Miranda			Rheumatology Center INC	Pembroke Pines, Florida, U	Collaborator	GRA
Michael	Guma			Riverside Medical Group	North Arlington, New Jerse	Collaborator	GRA
Ammar	Haikal			Riverside Medical Group	North Arlington, New Jerse	Collaborator	GRA
Sushama	Mody			Riverside Medical Group	North Arlington, New Jerse	Collaborator	GRA
Daric	Mueller			Shores Rheumatology PC	St. Clair Shores, Michigan, U	Collaborator	GRA
Arundathi	Jayatilleke			Temple University Hospital	Philadelphia, Pennsylvania,	Collaborator	GRA
JoAnn	Zell			University of Colorado	Boulder, Colorado, United S	Collaborator	GRA
Alison	Bays			University of Washington, Seattle	Seattle, Washington, Unite	Collaborator	GRA
Kathryn	Dao			UT Southwestern Medical Center	Dallas, Texas, United States	Collaborator	GRA
Fatemeh	Ezzati			UT Southwestern Medical Center	Dallas, Texas, United States	Collaborator	GRA

Supplemental Online Content: Nonauthor Collaborators

*Indicates required information. Only first name, last name, and suffix will appear in PubMed.

***First Name and Middle Initial(s)**	***Last Name**	***Suffix (eg, Jr, III)**	Academic Degrees	Institution	Location (city, state/province, country)	Role or Contribution, eg, chair, principal investigator	Group (if more than 1 Group listed in the byline) and/or Subgroup (eg, Steering Committee)
Deborah	Parks			Washington University Div of Rheumatology	St. Louis, Missouri, United S	Collaborator	GRA
David	Karp			UT Southwestern Medical Center	Dallas, Texas, United States	Collaborator	GRA
Guillermo	Quiceno			UT Southwestern Medical Center	Dallas, Texas, United States	Collaborator	GRA
